# Association between wildfire smoke exposure and parents’ adoption of protective behaviours: Exploring the role of objective and subjective smoke exposure

**DOI:** 10.1016/j.envc.2025.101362

**Published:** 2025-11-05

**Authors:** Catherine E. Slavik, Daniel A. Chapman, Stephanie E. Cleland, Perry Hystad, Ellen Peters

**Affiliations:** a Center for Science Communication Research, School of Journalism and Communication, University of Oregon, Eugene, OR, United States; b Faculty of Health Sciences, Simon Fraser University, Burnaby, BC, Canada; c Legacy for Airway Health, Centre for Lung Health, Vancouver Coastal Health Research Institute, Vancouver, BC, Canada; d College of Health, Oregon State University, Corvallis, OR, United States; e Department of Psychology, University of Oregon, Eugene, OR, United States

**Keywords:** Wildfire smoke, Smoke exposure, Children’s health, Risk perceptions, Exposure reduction, Protective behaviour, Air quality

## Abstract

Exposure to wildfire smoke poses a significant threat, particularly to children, even at low concentrations. Although several agencies monitor and disseminate air quality data, some parents rely on sensory cues to decide on protective behaviours, such as using air purifiers. We investigated relationships between objective smoke exposure measures (from air monitoring data), parents’ subjective perceptions of smoke exposure (perceived through sight or smell), and their protective behaviours during wildfire smoke events. We combined survey responses from 2086 parents in wildfire-prone regions of the western US and Canada with three and a half years of wildfire smoke data (2020–2023). Parents’ subjective perceptions of being exposed to smoke were associated with objective smoke exposure measures; however, subjective exposure was more strongly related to protective behaviours than objective exposure measures. Specifically, parents who perceived being exposed to wildfire smoke took, on average, more than one additional protective action (*b* = 1.11, *95% CI*: 0.92‒1.30), compared to those not who did not report smoke exposure. In comparison, every 10 μg/m^3^ increase of PM2.5 on smoke days predicted only a 0.23 increase in the number of protective actions adopted (*95% CI*: 0.06‒0.40). Exploratory analyses indicated non-linear relationships between objective smoke exposure and protective behaviours, with initial increases prompting more actions, plateauing at moderate levels, and rising again at higher exposure levels. As wildfire smoke can be harmful even when not visible or detectable by smell, smoke messaging should better connect objective air quality data like the Air Quality Index with parents’ subjective perceptions of wildfire smoke.

## 1. Background

Climate change and human activities have significantly accelerated wildfire activity globally, leading to longer, more frequent, and more intense wildfire seasons (Pausas and Keeley, 2021). In the fire-prone regions across the Western United States (US) and Canada, exposure to wildfire smoke poses a significant and growing threat to public health (Stowell et al., 2021; Yao et al., 2020). Wildfire smoke contains many air pollutants including particulate matter, toxic gases, and carcinogens. In recent years, up to half of the fine particulate matter (PM2.5) in the Western US can be attributed to wildfires, compared to less than 20 % a decade ago (Burke et al., 2021). As wildfire smoke can travel long distances, it can significantly degrade both local and regional air quality, potentially leading to serious health effects such as asthma exacerbation, heart failure, and impacts on cognitive and mental health (Mirabelli et al., 2022; Reisen et al., 2015). Exposure to PM2.5 from wildfire smoke cannot be controlled using the regulatory mechanisms that have been effective for other air pollutants, and it also appears to be more toxic to health than PM2.5 from other sources (e.g., traffic-related emissions) (Aguilera et al., 2021; Burke et al., 2023). Therefore, managing air quality risks during wildfire smoke episodes is crucial to safeguarding public health in the face of increasing wildfires.

Children appear to be especially sensitive to wildfire smoke due to their physiology and behaviours. For example, compared to the average adult, children spend more time outdoors, breathe more air relative to their bodyweight, have lungs that are still developing, and experience deeper deposition of fine particles into their lungs (Holm et al., 2021; Schöllnberger et al., 2002). Exposure to wildfire smoke has been linked to both short-term and long-term health consequences in children, such as increases in respiratory hospitalizations and declines in lung function (Black et al., 2017; Leibel et al., 2020). Even healthy children can suffer serious health effects from exposure and health impacts can occur at levels below regulatory thresholds (Henderson et al., 2024; Rice et al., 2021). As a result, risk-mitigation strategies aimed specifically at reducing children’s exposures to wildfire smoke are needed.

In recent years, public health agencies have made considerable efforts to encourage parents to adopt protective actions that reduce children’s exposure to wildfire smoke (British Columbia Provincial Health Services Authority, 2023; US CDC, 2024). Low-cost behaviours like checking smoke levels using air quality indexes, staying indoors and avoiding strenuous physical activity outdoors, and wearing well-fitted respirators during wildfire smoke events can significantly reduce smoke exposure (Laumbach, 2019; Slavik et al., 2023). Other effective smoke-safe actions for children and families include using portable air purifiers at home, relocating to areas with less smoke, and visiting clean air shelters in schools or community centres with high-quality ventilation systems (Balmes, 2023; Xu et al., 2020).

Although actions can be taken to decrease children’s exposure to wildfire smoke, increasing parents’ adoption of protective measures can be a significant challenge. Some parents do not know where to find air quality information, which can recommend protective actions based on the level of smoke in the air (Slavik et al., 2025). Also, not all parents know which actions are effective at reducing their children’s exposure to wildfire smoke nor have the resources to act (Santana et al., 2021). For example, Burke et al. (2022) found that online searches for protective items such as air filters and air purifiers occurred more frequently among wealthier individuals in response to daily smoke concentrations. The adoption of smoke-safe actions is also driven in large part by people’s concerns about a health threat and their desire to protect themselves and their families from it (Duan and Bombara, 2023). Certain susceptible groups, such as parents or children with asthma, appear to perceive higher risks from wildfire smoke and are more likely to take actions to reduce exposure (Hano et al., 2020; McCarron et al., 2024). Therefore, parents’ adoption of smoke-safe actions is likely influenced by both *objective* and *subjective* measures of wildfire smoke exposure.

Research exploring how perceptions of environmental exposures influence behaviour change has found that the decision to take protective actions against a hazard often begins with environmental cues—such as sights, smells, or sounds—that signal the onset of a threat (Lindell and Perry, 2012). For example, people’s experiences seeing or smelling hazy wildfire smoke-filled skies often inform perceptions of personal wildfire smoke exposure (Castillo et al., 2024; Seale et al., 2023; Walsh et al., 2022; Williamson et al., 2022). These cues also influence perceptions of health risk from an environmental hazard, such as the likelihood of experiencing harm, and feelings about potential health impacts (e.g., dread, worry) (D’Evelyn et al., 2022; Gordon et al., 2012). This may explain why individual experiences with wildfire smoke appear to drive smoke risk perceptions and motivate the adoption of protective actions, such as relocating to areas with better air quality or staying indoors (Berlin Rubin and Wong-Parodi, 2022; Buchanan et al., 2024; McGee and Healey, 2022). It also suggests that failing to recognize the risk posed by wildfire smoke could prevent some parents from taking essential precautions.

Little is known about how closely people’s subjective perceptions of wildfire smoke exposure resemble more objective indicators of smoke exposure, such as air quality levels or the air quality index. Some research has found that self-reported wildfire smoke exposure measures using smell or visual range (i.e., how far you can clearly see) correlate well with objective measures of wildfire smoke, such as concentrations of particulate matter (Künzli et al., 2006; O’Neill et al., 2013). This finding contrasts with results from studies that have compared subjective and objective exposure to ambient air pollution (Reames and Bravo, 2019; Song and Kwan, 2023), which suggest that people tend to underestimate exposure to air pollution from other non-wildfire sources. This discrepancy between perceptions of exposure to wildfire smoke and other non-wildfire-related sources of ambient air pollution could be the result of wildfire smoke having more obvious and visible environmental cues. Indeed, in one study, parents discussed using the smell of smoke as a signal to take actions like keeping their kids indoors (Santana et al., 2021). However, relying on these cues has limits as the relationship between PM2.5 concentrations and health effects is nonlinear (Henderson et al., 2024). At lower concentrations, the risk of adverse health effects increases more steeply; as a result, low-level wildfire smoke exposures (often undetectable by sight or smell) can have a disproportionately larger negative impact on population health than more extreme exposures.

There are numerous ways to measure the amount of wildfire smoke in the air, including measurements of PM2.5 concentrations from air quality monitors and satellite-derived estimates of smoke plume density, but it remains unclear whether certain operationalizations of smoke concentrations are more or less associated with protective behaviours. These represent significant gaps in knowledge as the development of effective public health strategies hinges on understanding the potential discrepancies between people’s perceived exposure to wildfire smoke and ambient smoke concentrations. If people’s perceptions of exposure motivate behaviour change more than real-time measurements of wildfire smoke, health education campaigns may need to speak to parents’ beliefs about the threats associated with wildfire smoke rather than solely communicate objective air quality data. Nonetheless, the use of objective exposure measures, such as air quality indexes, remain essential for detecting smoke that may not be visible or detectable by smell. Conversely, if objective measures of smoke concentrations motivate protective actions more than subjective perceived exposure, strategies to increase parents’ awareness of air quality monitoring data may be needed. Thus, comparing objective and subjective measures could provide insights for effectively tailoring interventions and protecting children from wildfire smoke.

In this study, we combined three and a half years of objective wildfire smoke measurement data with data from a cross-sectional survey of 2100 parents living in four wildfire-prone jurisdictions in the western US and Canada: California, Oregon, Washington, and British Columbia. These data were used to explore associations between objective measures of prior wildfire smoke exposure, subjective measures of prior wildfire smoke exposure, and other smoke perception measures. In the regions studied, many indoor environments lack air conditioning and mechanical ventilation systems, leaving them vulnerable to smoke infiltration and increasing the likelihood of exposure to wildfire smoke indoors (Lee et al., 2024; Liang et al., 2021; Xiang et al., 2021). Therefore, we conceptualize PM2.5 concentrations on smoke days as an indicator of wildfire smoke exposure and refer to these characterizations of ambient PM2.5 during wildfire smoke events as *objective* wildfire smoke exposure measures, while recognizing their limitations in capturing individual-level exposures. We also investigated relationships between subjective and objective wildfire smoke exposure and the adoption of smoke-protective actions, while controlling for various sociodemographic characteristics (education, pre-existing health conditions, income, gender, etc.). Additionally, we explored whether there are non-linearities in the relationship between objective smoke exposure and smoke-protective actions taken. Throughout our analyses, we examined how different operationalizations of objective wildfire smoke exposure (e.g., total number of smoke days versus average PM2.5 concentrations) are associated with subjective smoke exposure and the adoption of smoke-safe actions.

This research provides novel insights into several important areas. First, we are not aware of any prior research investigating the effects of subjective versus objective wildfire smoke exposure on people’s adoption of exposure-mitigating actions. Second, our use of multiple objective wildfire smoke exposure measures in our analyses provides insights into which measures serve as the most relevant predictors of smoke-safe actions. Finally, our exploration of potential non-linear effects of objective exposure on actions taken provides preliminary evidence that varying wildfire smoke thresholds may influence individuals’ behaviours in a non-linear fashion. These insights are important given increasing exposures to wildfire smoke across the globe.

## 2. Methods

### 2.1. Data sources

#### 2.1.1. Survey data

A sample of *N* = 2100 parents/guardians of children 18 years or younger were recruited between July and August of 2023 to take part in an experiment and survey on attitudes toward wildfire smoke. Experimental results from this study have been published separately (Slavik et al., 2024). All survey measures reported in the current paper were asked prior to any of the experimental components of the study. The median completion time was 13.64 min. To be eligible to participate, participants had to check a box on a consent form, be aged 18 years and over, and have at least one child aged 18 years or younger. We recruited participants from four jurisdictions in the US and Canada that have experienced significant wildfire smoke events in recent years: Washington, Oregon, California, and British Columbia. Canada-based participants were recruited using the panel provider Leger: 1054 participants from British Columbia met the study’s eligibility criteria and clicked the link to the survey, of whom 500 provided complete high-quality responses (i.e., consented to participate, passed two attention checks, and completed the study in at least 1/3rd of the median completion time or longer). US-based participants were recruited using the panel provider Centiment: 2941 participants from Washington, Oregon, and California met the study eligibility criteria and clicked the link to the survey, of whom 1600 provided complete high-quality responses using the same criteria. Sampling quotas were used to collect data that were roughly balanced by state/province, gender, and median household income.

This research received ethics approval from the University of Oregon and all participants provided informed consent prior to participating in the experiment. We retained 2086 participants in our analysis as these individuals provided postal or ZIP code information that could be validated and spatially linked to smoke data. The participants were relatively evenly distributed across the four jurisdictions: Oregon (*N* = 521), Washington (*N* = 538), California (*N* = 535), British Columbia (*N* = 492).

#### 2.1.2. Smoke exposure data

Daily smoke plume data and their associated densities (light, medium, and heavy) were downloaded from National Oceanic and Atmospheric Administration’s Hazard Mapping System (HMS) Fire and Smoke Product for 2020 to 2023 (National Oceanic and Atmospheric Administration, 2024). A given day in a postal/ZIP code was identified as a “smoke day” if there was any smoke plume observed overhead. The density of a “smoke day” was defined as the maximum density observed in a postal/ZIP code on a given day. The HMS Fire and Smoke Product includes only smoke plumes from vegetation fires, whereas smoke from other sources of fire is screened out (e.g., gas flaring, structural fires, etc.) (National Oceanic and Atmospheric Administration, n.d.). Additionally, PM2.5 observations were downloaded from the AirNow monitoring network (AirNow, 2024), which combines air quality data provided by government partners in the US and Canada. Hourly data for stations located in Oregon, Washington, California, and British Columbia were downloaded for 2020 to 2023 and aggregated to daily averages. Daily average PM2.5 observations were interpolated to postal/ZIP code centroids using thin-plate spline regressions for each day. A 10-fold cross-validation of the interpolated estimates indicated relatively good performance: an R^2^ of 0.67, with 87.8 % of estimates within 5 μg/m^3^ of the observed data, for peak smoke season estimates (June--September). To account for the fact that participants had not yet experienced a full year of smoke exposure in 2023 at the time of study recruitment, we only retained smoke data up to the date that each participant started their survey.

### 2.2. Dependent measure

#### 2.2.1. Smoke-Safe actions to mitigate exposure

The dependent measure was a composite score of actions participants reported having taken to reduce wildfire smoke exposure. The composite measure consisted of six yes/no items. First, participants were asked “During previous wildfire seasons, did air quality affect the decisions you made about having your child/children participate in outdoor activities (e.g., sports, recreation, leisure, or running errands)?”. Response options included “yes” (*n* = 1608; 77 %), “no” (*n* = 437; 21 %), and “don’t know” (*n* = 41; 2 %). This item was then re-scored by aggregating the “don’t know” and “no” responses together to form a binary measure. Next, participants were given the following prompt: “During previous wildfire seasons, have you ever done any of the following during periods of wildfire smoke in your community?” They were given the following five actions to respond to (yes/no), including the following: “checked air quality levels in your area before going outside” (Yes: *n* = 1648; 79 %), “cancelled or rescheduled outdoor activities” (Yes: *n* = 1590; 77 %), “relocated outdoor activities to area with better air quality” (Yes: *n* = 1194; 58 %), “kept windows and doors closed to keep smoke out” (Yes: *n* = 1885; 91 %), and “used a portable air purifier to improve indoor air quality” (Yes: *n* = 1132; 55 %). Responses to these six binary items were then summed together such that higher scores reflect more actions taken. The scale could range from 0 (none) to 6 (all), mean (*M)* = 4.34, standard deviation (*SD*) = 1.65. The composite had a Cronbach’s alpha (a measure of scale reliability) of 0.71.

### 2.3. Independent measures

#### 2.3.1. Demographics

The following demographic characteristics were measured in the survey and included as independent variables in our regression models: education, health conditions, family income, gender, and age. Education was analysed using a binary measure based on whether participants had a Bachelor’s degree or higher, or not. To measure health conditions, participants were asked whether they or their child/children had one or more of a list of health conditions that may be impacted by wildfire smoke. These included: asthma, chronic obstructive pulmonary disease, hypertension or high blood pressure, other heart disease, type II diabetes, metabolic syndrome or obesity, allergies related to the upper respiratory tract, eyes, and ears, or other chronic disease. Participants were also asked to provide their household family income from a list of 17 brackets. Gender was analysed using three categories: man, woman, and other, which included participants who had responded either transgender, non-binary/non-conforming, or “other”. Lastly age was analysed as a continuous variable.

#### 2.3.2. Wildfire smoke perception measures

The following three wildfire smoke perception measures were measured in the survey and included as independent variables in our regression models: subjective past smoke exposure, perceived future susceptibility to wildfire smoke, and worry/concern about wildfire smoke. Subjective smoke exposure was measured with the following question: “During previous wildfire seasons, was your community impacted by wildfire smoke that you could see or smell?” Participants could answer “yes” (*n* = 1797), “no” (*n* = 237), or “don’t know” (*n* =52). The “don’t know” responses were grouped with the “no” responses for analysis purposes. Perceived susceptibility to wildfire smoke was measured with the following question: “Do you believe that a major wildfire event will impact the air quality where you live in the next two years?” Response options included “yes” (*n* = 1625), “no” (*n* = 174), and “unsure” (*n* = 287). The “unsure” responses were grouped with the “no” responses for analysis purposes. Worry/concern about wildfire smoke was measured using two items: “How worried or concerned are you about wildfire smoke?” and “If you or your family members had to spend the day outside, how worried or concerned would you be about the health risks of wildfire smoke”. Responses could range from 1 (not at all worried or concerned) to 7 (very worried or concerned). These two items were highly correlated, *r* = 0.71 [95 % CI: 0.69, 0.73], and thus a composite score was created by averaging the two items together, *M* = 4.64, *SD* = 1.65.

#### 2.3.3. Objective smoke exposure measures

Daily smoke plume data from NOAA’s HMS Fire & Smoke Product and PM2.5 observations from AirNow from 2020 to 2023 were used to construct nine objective smoke exposure measures used in our regression models. These included:

1. Total Number of Smoke Days: Number of smoke days (any density) with a smoke plume overhead between Jan 1, 2020 and the date respondent started survey. *M* = 209.88, *SD* = 34.55, Min = 107, Max = 326.
2. Total Number of Smoke Days of PM2.5 > 15 μg/m^3^: Number of smoke days (any density) where the daily average PM2.5 concentration exceeded 15 μg/m^3^, calculated between Jan 1, 2020 and the date respondent started survey. This threshold for PM2.5 was selected as it is the WHO air quality guideline for 24-hour average PM2.5 (World Health Organization, 2021). *M* = 46.89, *SD* = 25.26, Min = 8, Max = 157.
3. Total Number of Light Smoke Days: Number of light density smoke days calculated between Jan 1, 2020 and the date respondent started survey. Light density smoke plumes were obtained from NOAA’s HMS Fire & Smoke Product (Ruminski, n.d.). *M* = 123.46, *SD* = 13.34, Min = 79, Max = 170.
4. Total Number of Medium Smoke Days: Number of medium density smoke days calculated between Jan 1, 2020 and the date respondent started survey. Medium density smoke plumes were obtained from NOAA’s HMS Fire & Smoke Product (Ruminski, n.d.). *M* = 48.71, *SD* = 11.36, Min = 16, Max = 92.
5. Total Number of Heavy Smoke Days: Number of heavy density smoke days calculated between Jan 1, 2020 and the date respondent started survey. Heavy density smoke plumes were obtained from NOAA’s HMS Fire & Smoke Product Ruminski, n.d.). *M* = 37.70, *SD* = 19.85, Min = 3, Max = 148.
6. Total Number of 2-day Smoke Waves: Number of smoke waves where there were ≥2 consecutive days with a smoke plume (any density) and daily average PM2.5 exceeded 15 μg/m^3^ between Jan 1, 2020 and the date respondent started survey. This definition is similar to previous definitions of smoke waves (Casey et al., 2024). *M* = 32.20, *SD* = 5.32, Min = 19, Max = 47.
7. Total Number of 3-day Smoke Waves: Number of smoke waves where there were ≥3 consecutive days with a smoke plume (any density) and daily average PM2.5 exceeded 15 μg/m^3^ between Jan 1, 2020 and the date respondent started survey. *M* = 23.63, *SD* = 3.35, Min = 12, Max = 32.
8. Total Number of Smoke Days With AQI>100: Number of smoke days (any density) where the daily average PM2.5 concentration exceeded 35.5 μg/m^3^, which corresponds to when the US-AQI exceeds 100 (‘Unhealthy for Sensitive Groups’ category or above), calculated between Jan 1, 2020 and the date respondent started survey. *M* = 16.06, *SD* = 10.78, Min = 1, Max = 78.
9. Average PM2.5 on Smoke Days: Average of daily PM2.5 on smoke days (any density) calculated between Jan 1, 2020 and the date respondent started survey. *M* = 16.06, *SD* = 4.17, Min = 4.34, Max = 36.22.

### 2.4. Statistical analysis

We first examined the extent to which each of our objective smoke exposure metrics were associated with participants’ self-reported past exposure to wildfire smoke. Separate models were run for each of the objective smoke metrics, except for the light/medium/heavy smoke variables, which were included in a single model, consistent with Buchanan et al. (2024). In each model, random intercepts were fit for each postal/ZIP code, and two controls were also included: average PM2.5 on non-smoke days (to adjust for other forms of air pollution), and the distance to the nearest monitoring station from the AirNow monitoring network (log km). Three quarters of the participants had a monitoring station within 5 km of their postal code/ZIP code and 23 % had one within their postal code/ZIP code. Each model of self-reported past exposure was a binary logistic multilevel regression model.

**Fig. 1.**
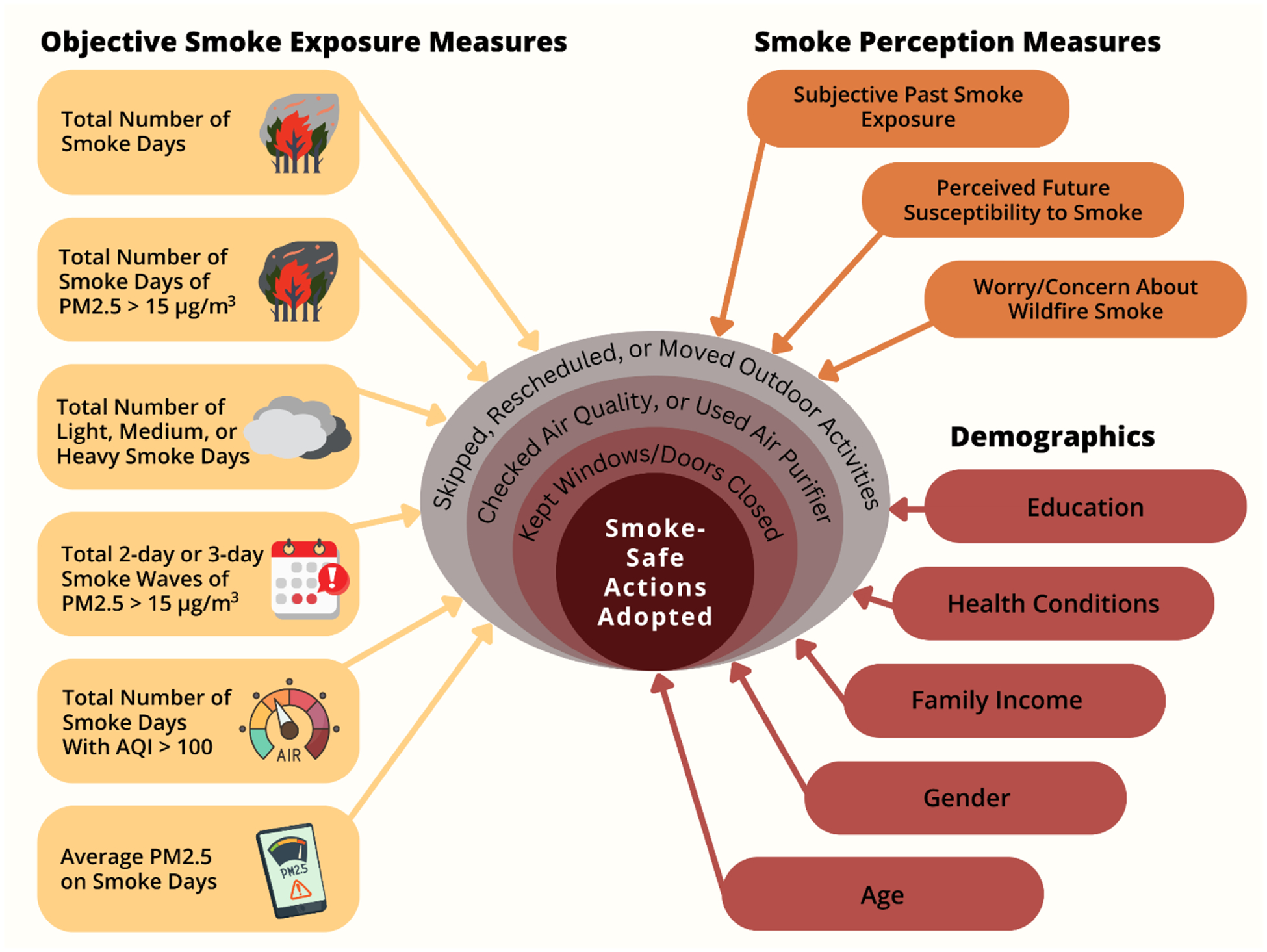
Framework for predicting the adoption of wildfire smoke protective actions. Six measures of wildfire smoke exposure, three measures of participants’ wildfire smoke perceptions, and a variety of demographic factors served as independent variables in this study.

Next, we performed a three-step modelling process to examine how objective and subjective smoke exposure measures, along with an ensemble of other survey measures (demographics, worry about wildfire smoke, perceived future susceptibility) predict self-reported actions taken to mitigate wildfire smoke. For a conceptual framework, see Fig. 1.

In Model Set 1, we only fit the objective smoke exposure measure and the two controls (average PM2.5 on non-smoke days and distance to nearest monitoring station). Sensitivity analyses re-ran the models without distance to nearest monitoring station included. In Model Set 2, we included just the subjective smoke exposure measure in the model. In Model Set 3, we added the objective and subjective measures simultaneously, and in Model Set 4 we added the full ensemble of predictors. Linear multilevel regression models were fit with random intercepts for postal/ZIP codes.

For all models described above, we deemed a model parameter to be statistically significant if the 95 % confidence intervals did not cross 1 in the case of odds ratios for binary outcome variables or did not cross 0 for continuous outcome variables.

We then performed exploratory non-linear regression models using a generalized additive modelling framework (Wood, 2017). Non-linearity was modelled using cubic regression splines, and we again fit the models with random intercepts for postal/ZIP code. As the non-linear models were exploratory in nature, we rely primarily on a descriptive interpretation of the data based on graphical depictions of the slopes. In the Online Supplement, we provide tables of the statistical summaries from the models (see Tables S9 and S10). Exploratory models breaking down the slopes for objective exposure by demographic characteristics were again fit using cubic splines in a generalized multilevel additive model. In this case, we fit separate non-linear slopes for each level of a demographic factor (e.g., separate slopes for men and women). Results of these models are reported in the Online Supplement as well (see Fig. S1–S5; Tables S11–S15).

When reporting the results, we report standardized (*M* = 0, *SD* = 1) regression coefficients for the objective measures in the main text and supply unstandardized coefficients in the Online Supplement. To aid in model interpretability of the unstandardized coefficients, we transformed several variables prior to fitting the models. Objective smoke exposure metrics which were quantified in terms of days (e.g., total smoke days, total light/medium/heavy smoke days) were divided by seven so that 1-unit changes in these measures correspond to 1-week changes in the number of smoke days. Average PM2.5 on smoke (and non-smoke) days were transformed from 1 μg/m^3^ increments to 10 μg/m^3^ increments so that a 1-unit change in the measure corresponds to a 10 μg/m^3^ increment change in average PM2.5. The 10 μg/m^3^ concentration for PM2.5 is a common benchmark used in air pollution research as it aligns with the World Health Organization’s former air quality guidelines (revised in 2021) (World Health Organization, 2021), allowing for comparability across various studies. The total number of two and three-day smoke waves were kept on their original scale. The remaining survey predictors on a metric scale (family income, age, and worry about wildfire smoke) were z-scored (*M* = 0, *SD* = 1) so that 1-unit changes reflect 1 SD changes in the predictors. The remaining variables are binary classifiers, which were not transformed and were entered as dummy codes into the models.

All data analysis was performed in R version 4.4.1 (R Core Team, 2024). The logistic and linear multilevel models were fit using the lme4 package (Bates et al., 2015). Statistical summaries of parameter estimates and confidence intervals were generated using the parameters package (Lüdecke et al., 2020). Effects plots were generated using ggplot2 and the sjPlot packages (Lüdecke, 2024; Wickham et al., 2021). The exploratory non-linear models were performed using mgcv and gamm4 packages (Wood and Scheipl, 2020; Wood, 2017), with plotting performed using the gratia package (Simpson, 2024). To facilitate computational reproducibility, all data and analysis code necessary to recreate the results reported in each model set can be found on the open science framework: https://osf.io/sytgp/files.

## 3. Results

### 3.1. Participant characteristics

Of the 2100 surveyed participants, 2086 had a self-reported postal/ZIP code in the four jurisdictions that could be spatially linked to the objective smoke exposure data. The study sample of *N* = 2086 had a mean age of 39 (*SD* = 9). More women (*n* = 1187) than men (*n* = 872) participated, while 1 % (*n* = 24) identified as another gender. Just under half of the sample had a Bachelor’s degree or higher (*n* = 954). When participants were asked whether they or their child/children had one or more health conditions that may be impacted by wildfire smoke (e.g., asthma), over half of participants (*n* = 1234) reported one or more relevant conditions. The median family income was between $80–89,999. Table S1 in the Online Supplement provides the demographic breakdown of the sample by quartiles of average PM2.5 on smoke days and by subjective smoke exposure. Participants who lived in areas with higher quartiles of average PM2.5 on smoke days tended to skew female, be less educated, have more health conditions, live in Oregon, and have a lower income. Age was approximately equivalent across the quartiles.

### 3.2. Associations between objective and subjective wildfire smoke exposure

We examined the association between nine objective measures of wildfire smoke exposure and participants’ subjective reports of past smoke exposure to identify how related each objective measure was to participants’ likelihood of reporting past wildfire smoke exposure. As shown in Fig. 1, objective exposure measures included, for example, the total number of days with smoke (any density) experienced by the study participant during the study period, days with smoke where PM2.5 concentrations exceeded 15 μg/m^3^ (the WHO’s guideline for daily air quality), and days with smoke where PM2.5 concentrations pushed the Air Quality Index (AQI) above 100 indicating conditions are “Unhealthy for Sensitive Groups.” We also assessed days by the density of smoke (light, medium, or heavy) based on categorization of smoke plumes in NOAA’s HMS smoke product. Lastly, we considered multi-day exposure events as another objective measure, based on the number of 2-day and 3-day smoke waves with PM2.5 levels above 15 μg/m^3^. All measures of objective smoke exposure were for 2020 until the day of the survey in 2023.

**Fig. 2.**
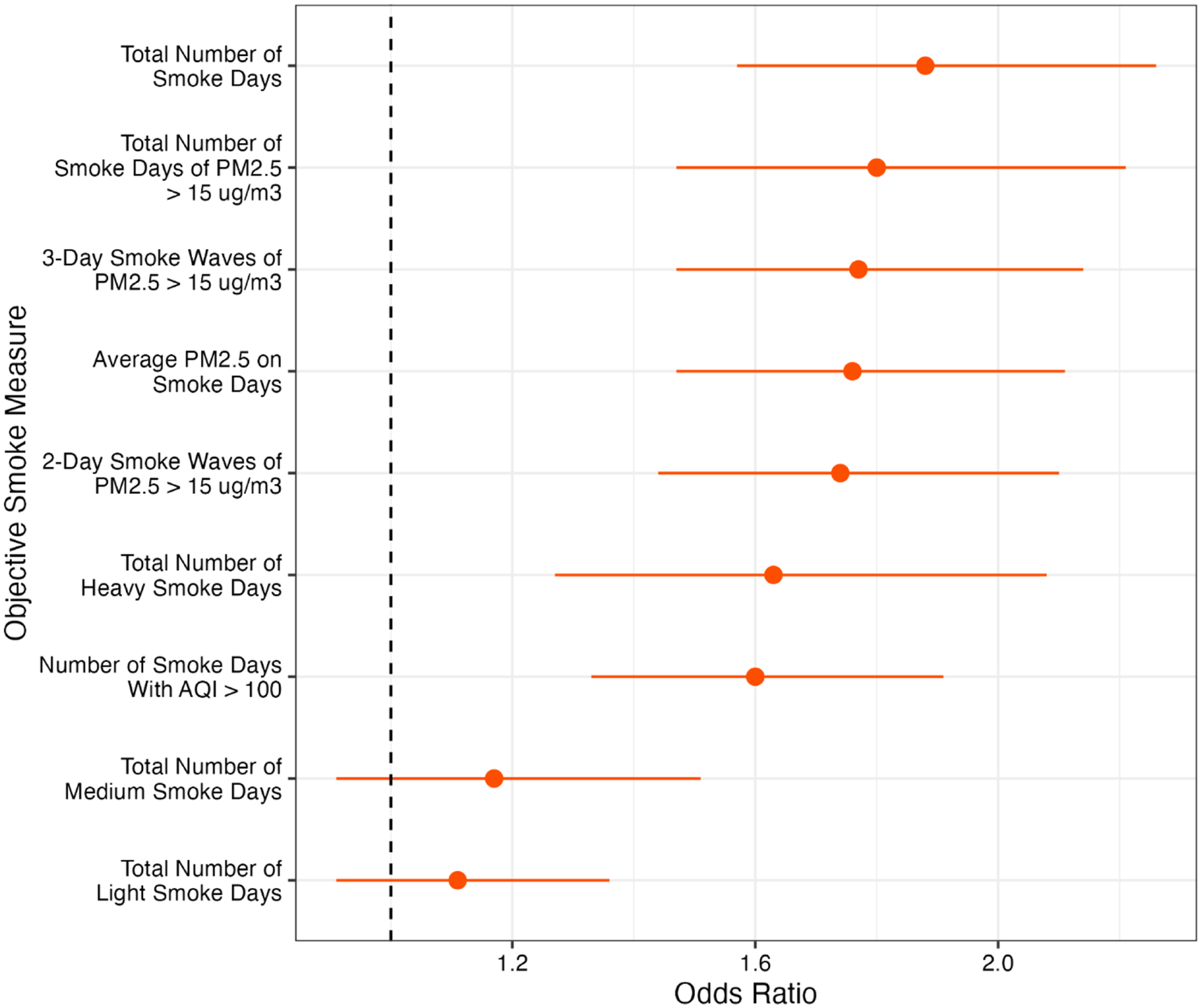
Effects of various objective smoke measures on parents’ subjective past exposure to wildfire smoke. All smoke measures were z-scored (*M* = 0, SD = 1) to allow comparison of coefficients across models. The vertical dashed line is placed at an odds ratio of 1, which indicates no effect. Odds ratios were derived using binary logistic multilevel regression models that included random intercepts for postal/ZIP code as well as the following controls: average PM2.5 on non-smoke days (z-scored) and distance to nearest monitoring station (log km; z-scored). All objective smoke measures were calculated from 2020 until the survey date in 2023. In the Online Supplement, Table S2 provides the full set of standardized regression coefficients and Table S3 provides unstandardized versions.

Logistic regression models were fit to the model to examine the extent to which each objective measure (entered as predictors in separate models) was associated with our binary subjective exposure measure (see Methods section for additional model information). To enable cross-measure comparisons, in these models we standardized the objective smoke measures each to have a mean (*M*) of 0, and standard deviation (*SD*) of 1 (see Methods section for the raw descriptive statistics for these objective smoke measures). We found significant associations between all objective smoke measures—except for the total number of light and medium density smoke days—and parents’ subjective past exposure (see Fig. 2 for a visual depiction; see Table S2 for standardized regression coefficients). For example, a 1 *SD* change in the total number of smoke days (35 days) was associated with a 1.88 increase in the odds of reporting smoke exposure (*OR* = 1.88, *95 % CI*: 1.57–2.26). A 1 *SD* change in the total number of smoke days exceeding 15 μg/m^3^ (25 days) was associated with a 1.80 increase in the odds of reporting smoke exposure (*OR* = 1.80, *95 % CI*: 1.47–2.21). Significant associations were also found for 3-day smoke waves where PM2.5 exceeded 15 μg/m^3^ (*OR* = 1.77, *95 % CI*: 1.47–2.14) and average PM2.5 on smoke days (*OR* = 1.76, *95 % CI*: 1.47–2.11). See the Online Supplement for the full summaries of the models (Table S2). Distance to the nearest air quality monitoring station did not influence the association between objective and subjective past exposure. Average PM2.5 on non-smoke days did not have a consistent effect across models. All models were re-fit excluding the distance to the nearest monitoring station variable. There was no substantive change to the results when this variable was removed, and therefore we do not consider these alternative models further.

For ease of interpretation, we also present the unstandardized model output in the Online Supplement displaying odds ratios in unit increases relevant to epidemiological research. For example, the effect of average PM2.5 on smoke days was such that every 10 μg/m^3^ increase in average smoke day PM2.5 was associated with participants being nearly 4 times more likely to self-report previous smoke exposure (*OR* = 3.89, *95 % CI*: 2.52–6.00). See the Online Supplement for the full model summaries (Table S3).

### 3.3. Associations between objective and other smoke perception measures

In addition to looking at subjective past smoke exposure (as detailed in the prior section), we also examined the relationship between the objective smoke exposure measures and two other smoke perception measures: perceived future susceptibility to smoke impacts and worry about wildfire smoke. As with the subjective exposure measure, we found significant associations between all objective smoke measures—except for the total number of light and medium density smoke days—and perceived future susceptibility and worry. For the complete list of standardized and unstandardized regression coefficients for each of the objective smoke measure models, see Tables S2 and S3 in the Online Supplement.

**Fig. 3.**
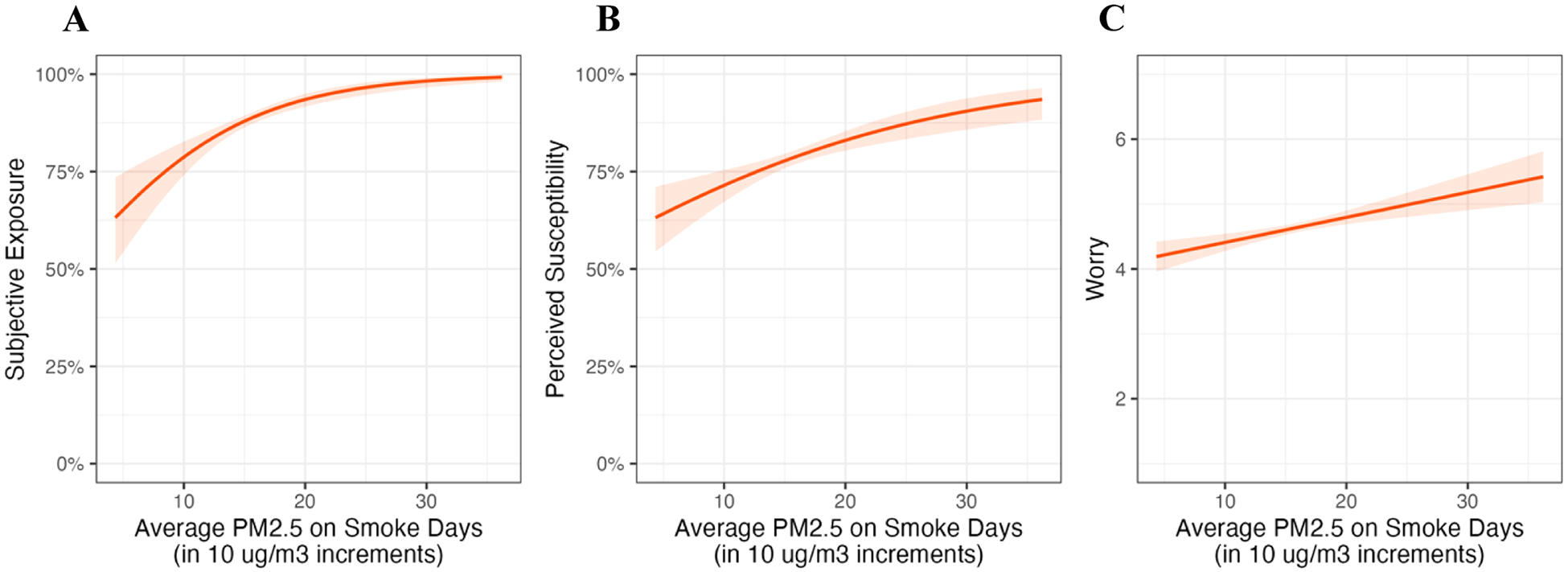
Effects of average PM2.5 on smoke days on parental smoke perceptions. Three smoke perception measures included (A) subjective past smoke exposure (binary), (B) perceived future susceptibility to smoke impacts (binary), and (C) worry about wildfire smoke (7-point scale). *Note*. Results derived from binary logistic and linear multilevel models. Covariates in the model included average PM2.5 on non-smoke days and distance to the nearest monitoring station. Postal/ZIP code was included as a random intercept parameter in each model. Predicted probabilities from the model are displayed along with shaded 95 % confidence intervals. Standardized and unstandardized coefficients for each model can be found in the Online Supplement (Tables S2 & S3).

To highlight an example, we plot the relationship between average PM2.5 on smoke days and all three smoke perception measures in Fig. 3. Using the standardized version of the PM2.5 measure, results indicated that a 1 SD increase in the average concentration of PM2.5 on smoke days (4.17 μg/m^3^) were positively associated with perceived future susceptibility (*OR* = 1.32, *95 % CI*: 1.16–1.50), and worry about wildfire smoke (*b* = 0.16, *95 % CI*: 0.08–0.24). For ease of interpretation, we also present the unstandardized model output in the Online Supplement (Table S3) displaying associations in unit increases relevant to epidemiological research.

### 3.4. Predicting smoke-safe actions from objective and subjective exposure

Next, we examined whether objective or subjective wildfire smoke exposure was more strongly associated with protective actions taken. We started with objective measures only. In Model Set 1, we fit linear multilevel models entering the objective smoke measures (in separate models) as predictors of total number of protective smoke-safe actions taken, including PM2.5 on non-smoke days and distance to the nearest monitoring station as controls (Table 1). We report the focal coefficients in the main text; the full model summaries of all coefficients can be found in the Online Supplement (see Table S4).

Results indicated that each objective smoke measure—except for light and medium density smoke days—had a small but significant effect on the number of smoke-safe actions taken. In each model, a 1 *SD* increase in the objective exposure measure was associated with a 0.11–0.21 unit increase in the number of actions taken. For ease of interpretation, we also present the unstandardized model output in the Online Supplement (See Table S5). For example, results indicated that every 10 μg/m^3^ increase in average PM2.5 on smoke days predicted a 0.49 increase in the number of smoke-safe actions adopted. Thus, every 20 μg/m^3^ increase in average PM2.5 concentrations on smoke days would lead to about one additional smoke protective action being adopted.

In Model Set 2, we fit a linear multilevel model entering subjective smoke exposure as a predictor of total number of protective smoke-safe actions taken, again including PM2.5 on non-smoke days and distance to the nearest monitoring station as controls (Table 1). We report the focal coefficients in the main text; the full model summaries of all coefficients can be found in the Online Supplement (see Tables S4 and S5). On its own, subjective exposure had a coefficient of 1.71 (*95 % CI*: 1.51‒1.90), indicating that those who reported past exposure reported taking 1.71 more actions on average than those who did not report past exposure.

In Model Set 3, we included the subjective smoke exposure and each objective smoke exposure measure as co-predictors in separate models (as was done in Model Set 1) predicting the total number of smoke-safe actions taken (Table 1). These models again included PM2.5 on non-smoke days and distance to the nearest monitoring station as control variables. The focal coefficients are reported in Table 1, and the full model summaries can be found in the Online Supplement (see Table S4).

Results indicated that, after accounting for the small effects of the objective exposure measures, subjective exposure had a significant, large effect on the number of actions taken. In each model, those with prior subjective exposure were approximately 1.66 to 1.70 units higher on number of actions taken than those without prior subjective exposure. In fact, each of the objective exposure measures were reduced to non-significance in the presence of subjective exposure, except for average PM2.5 on smoke days (Objective indicator G, Table 1). In this model, participants who reported prior subjective exposure to wildfire smoke took nearly twice as many smoke-safe actions (*b* = 1.66, *95 % CI*: 1.47–1.86) compared to those who did not report subjective exposure. We also fit the models using more readily interpretable unit increases as opposed to a 1 *SD* increase. For example, every 10 μg/m^3^ increase of PM2.5 on smoke days was associated with a 0.27 increase in the number of smoke-safe actions adopted (See Table S5).

While we did z-score the objective smoke measures, it is true that a binary measure such as our subjective smoke exposure measure may still not be directly comparable to a z-scored continuous measure, making coefficient comparisons difficult. Therefore, we also performed a follow-up analysis in which we aimed to quantify the predictive performance of each measure using the Akaike Information Criterion (AIC) (Akaike, 1998). AIC is a measure intended to quantify the anticipated out-of-sample predictive performance of a model. Models with a smaller AIC indicate a better fit than those with larger AIC values. Table S6 in the Online Supplement provides AIC values for each model. Across the models, each objective smoke exposure measure had a similar AIC value, while the subjective smoke exposure had the lowest AIC value. These results further suggest that the subjective exposure measure may be a better predictor of actions taken compared with the objective smoke measures.

**Fig. 4.**
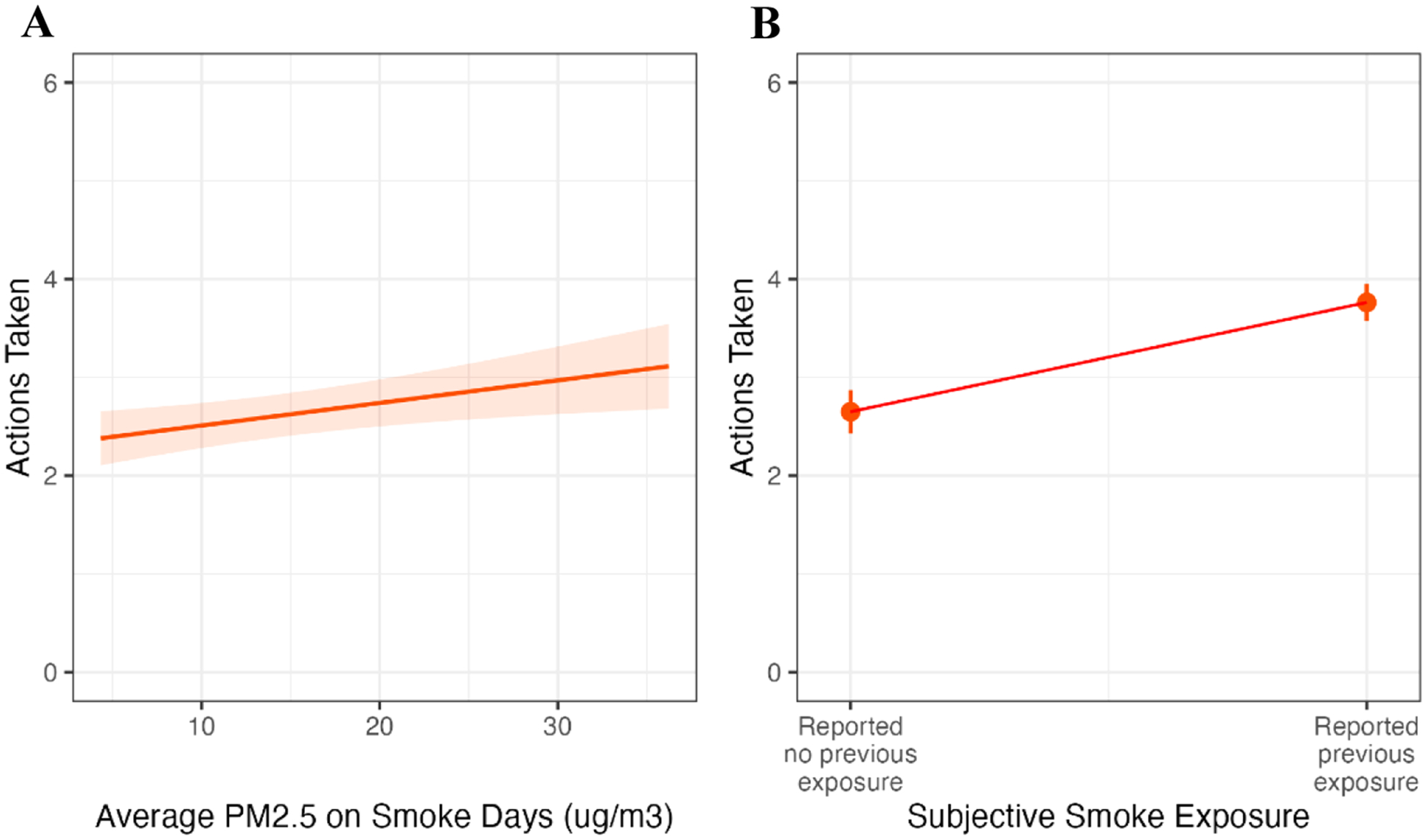
Predicted effects of objective and subjective smoke exposure on smoke-safe actions taken. (A) Effects of average PM2.5 on smoke days on smoke-safe actions taken. (B) Effects of subjective smoke exposure on smoke-safe actions taken. Results derived from a linear multilevel regression model, where average PM2.5 of smoke days and subjective smoke exposure were entered alongside an ensemble of covariates. Error bars reflect 95 % confidence intervals around the model predictions.

Next, we fit a series of Model Set 4 s (one for each objective exposure measure) where we entered our full ensemble of demographic and other covariates alongside the objective and subjective exposure measures (see Table S7 for standardized and Table S8 for unstandardized coefficients). As with Model Set 3, when entering in all variables, the only objective smoke measure which remained significant was average PM2.5 on smoke days. Subjective smoke exposure remained a large, significant predictor of the number of smoke-safe actions taken. Despite lack of variance in the binary subjective smoke exposure measure, its standardized coefficient was about eight times larger than that for average PM2.5 (Table 1). Fig. 4 plots the predicted effect of average PM2.5 on smoke days, and subjective smoke exposure, on the number of smoke-safe actions taken.

**Fig. 5.**
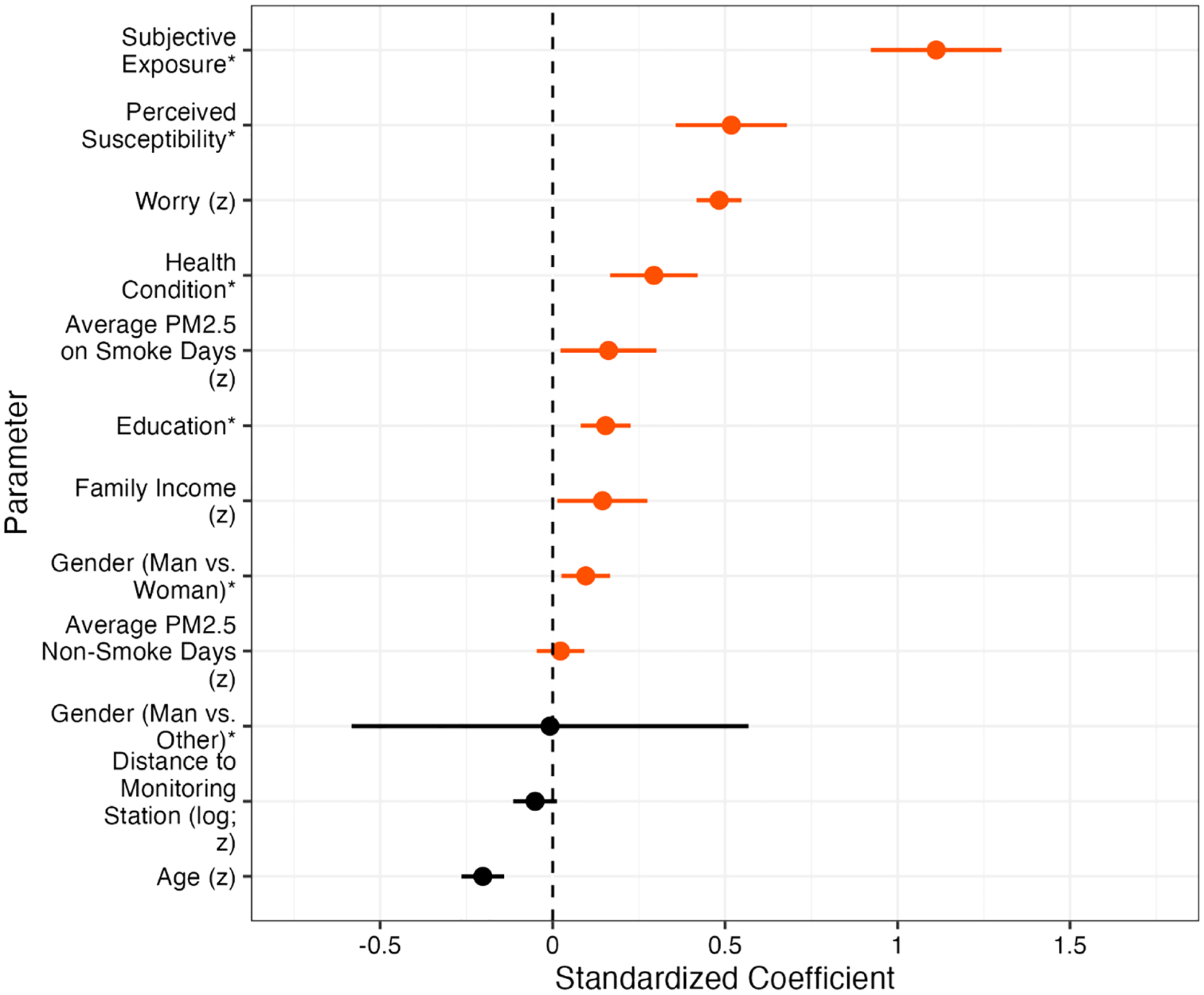
Forest plot of all regression coefficients on the number of smoke-safe actions taken. Points reflect standardized regression coefficients and error bars are 95 % confidence intervals. Values with positive coefficients are in orange, and values with negative coefficients are in black. * = binary variable. Values marked with (z) denote z-scored variables (*M* = 0, SD = 1).

Fig. 5 plots the standardized parameter estimates for each variable in the model with the full ensemble of covariates, subjective exposure, and average PM2.5 on smoke days as the objective exposure. There was a significant effect of perceived susceptibility, indicating that those who perceive themselves as more susceptible to smoke impacts were more likely to report protective actions taken. There was also a similar positive effect of worry about smoke on actions taken. Those with a Bachelor’s degree or greater, higher income, and reporting relevant health conditions (in themselves or their child/children) all reported significantly more actions taken. Women reported more actions taken than men, and younger parents reported more actions taken than older parents. These results are highly consistent across the other models, in most cases to the hundredths of thousandths decimal place. In the Online Supplement, we provide a table which displays the coefficients for each model alongside one another to enable comparison across models (see Table S7 for standardized and Table S8 for unstandardized coefficients).

### 3.5. Non-linear effects of objective smoke exposure on actions taken

**Fig. 6.**
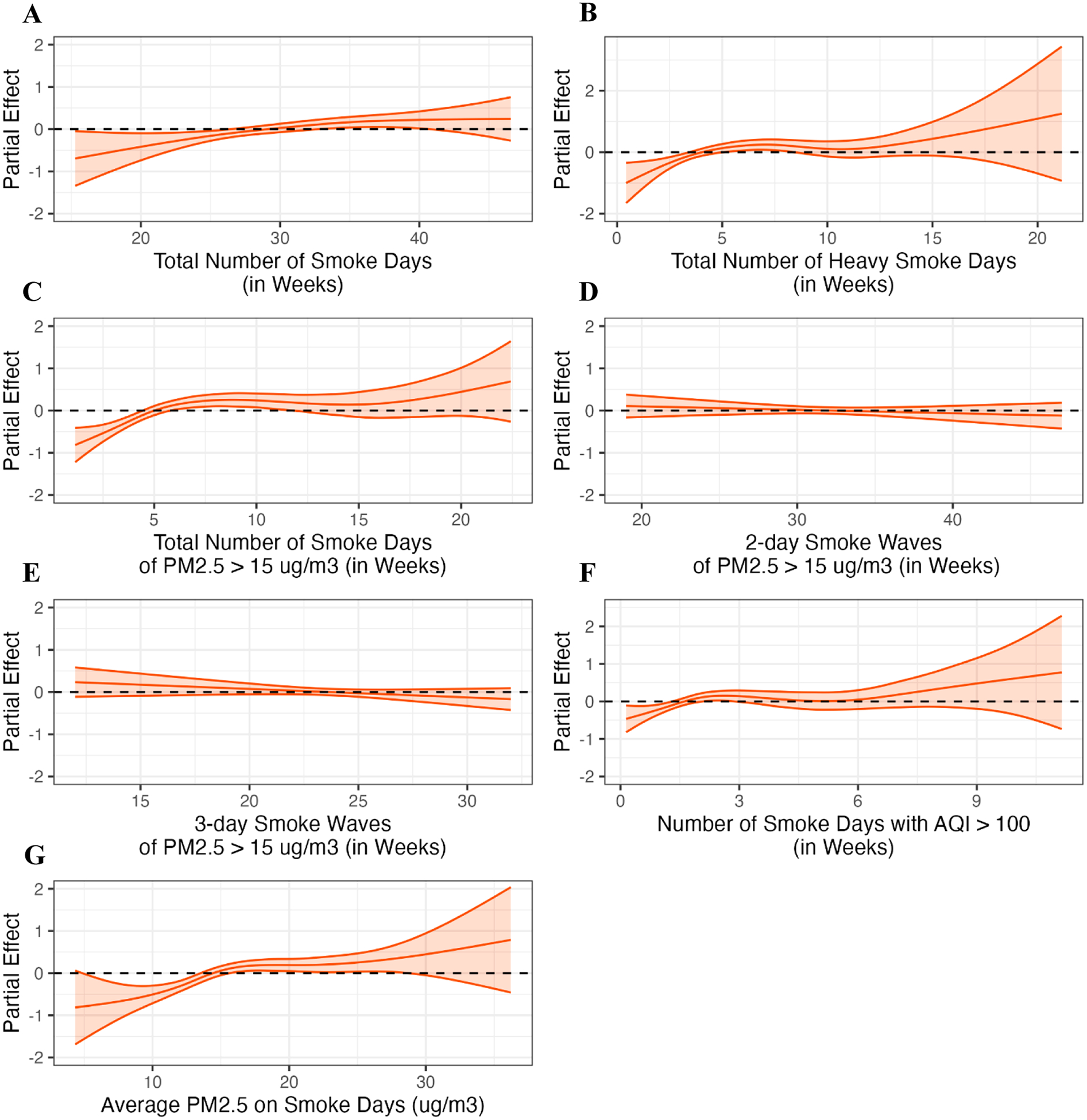
Non-linear multilevel models predicting smoke-safe actions taken from each objective smoke measure. (A) Total number of smoke days (any density). (B) Total number of heavy smoke days. (C) Total number of smoke days of PM2.5 > 15 μg/m3. (D) Number of smoke waves with ≥2 consecutive days with a smoke plume (any density) and daily average PM2.5 exceeded 15 μg/m3. (E) Number of smoke waves with ≥3 consecutive days with a smoke plume (any density) and daily average PM2.5 exceeded 15 μg/m3. (F) Total number of smoke days with AQI>100. (G) Average of daily PM2.5 on smoke days. Partial effect (y-axis) refers to increases and decreases in the number of actions taken relative to the mean (i.e., 0), adjusted for covariates in the model. Cubic regression splines were fit for each objective smoke measure. As controls, average PM2.5 on non-smoke days and distance to the nearest monitoring station were included. Average PM2.5 on non-smoke days was also fit using a cubic regression spline. Neither light nor medium smoke days had significant effects in the model and are not displayed here. Shaded areas and outer lines denote 95 % confidence intervals. In further exploratory models, we examined how the nonlinear slopes varied across the levels of the five demographic characteristics; for example, we allowed separate nonlinear slopes for men and women, and older adults compared with younger adults. Results indicated that the slopes were similar across the various demographics, and where different trends exist, they were within the margin of error. Moreover, where evidence for nonlinearities appeared in the tails, there tended to be much fewer datapoints, increasing the uncertainty in these estimates. Therefore, we only present these models in the Online Supplement for interested readers and do not consider them further in text (Tables S11–S15).

As an exploratory analysis to ascertain potential smoke thresholds that might drive behaviour, we refit each objective smoke measure alongside the two controls (i.e., average PM2.5 on non-smoke days and distance to the nearest monitoring station) as predictors of smoke-safe actions taken using cubic regression splines in generalized additive models. Since this analysis was exploratory and aimed to examine the nonlinear effects of objective measures on the number of smoke-safe actions taken, we did not include the full range of smoke perception and demographic variables as covariates in the models. Fig. 6 displays the effects of each objective measure on actions taken. ‘Partial effects’ are plotted on the y-axis. These represent the effect of x on the outcome, conditional on the other parameters. These models mean-centre effects for identifiability reasons, and thus the partial effect is centred around zero on the dependent variable (i.e., the mean of actions taken). Thus, values higher than zero reflect positive changes above the average, and values lower than zero reflect negative changes below the average. The Online Supplement contains the full statistical summaries (see Table S9 and S10). There were significant non-linear effects for total number of smoke days, total number of heavy smoke days, total number of smoke days exceeding 15 μg/m^3^, number of smoke days where AQI exceeded “unhealthy for sensitive groups”, and average PM2.5 on non-smoke days. These followed a mostly linear pattern (see, e.g., total number of smoke days). However, there was some evidence of non-linearities such that initial increases in exposure led to more actions taken, followed by a plateau in actions taken at moderate exposure levels, increasing again at high levels of exposure. There was, however, a considerable degree of uncertainty in the estimates at the highest levels of exposure, suggesting more data are needed to produce precise estimates of the non-linear effects.

## 4. Discussion

In this study, we combined responses from a cross-sectional survey of parents living in the western US and Canada with three and a half years of wildfire smoke data to explore the relationship between subjective and objective wildfire smoke exposure measures and to investigate their associations with the adoption of smoke-protective actions. We found that most of the nine objective smoke measures were associated with parents’ subjective (self-reported) exposure to wildfire smoke. These objective exposure measures were also positively associated with two other smoke perception measures: participants’ worry about wildfire smoke and their perceived susceptibility to future smoke events.

However, our analysis revealed that *subjective* exposure to wildfire smoke may be more strongly associated with the adoption of parents’ protective actions than any of the nine *objective* smoke exposure measures studied (Table 1). These results suggest that, irrespective of whether subjective perceptions of wildfire smoke exposure align with ambient PM2.5 concentrations, such perceptions likely play a crucial role in motivating parents to take protective measures during wildfire smoke events. These findings also support the need to evaluate current risk communication strategies intended to encourage the adoption of protective measures against wildfire smoke. While risk communications tend to focus on informing the public about smoke levels in the air—an objective exposure measure—our results suggest that this strategy may not drive perceptions adequately.

Only one out of the nine objective exposure measures emerged as a consistent significant predictor of smoke-safe actions taken—average PM2.5 on smoke days—when also accounting for subjective exposure and the other covariates. This finding offers important insights into how someone’s typical experiences with air quality on smoky days may impact perceptions of exposure and lead to behaviour change. Despite examining multiple other objective exposure measures, such as cumulative smoke days and the number of smoke waves, our results suggest that people’s perceptions of wildfire smoke exposure appear to be more informed by the *severity* of smoke (i.e., average concentrations) as opposed to the *frequency* or *duration* of wildfire smoke events. Going forward, it will be important to continue to evaluate multiple wildfire smoke metrics to better understand which types of exposures are most relevant for different outcomes, such as behaviour change versus specific health effects.

Interestingly, participants’ likelihood of reporting worry and their perceived susceptibility to experiencing future wildfire smoke events also increased with higher average PM2.5 concentrations on smoke days (Fig. 3). These results would indicate that experiences with more severe wildfire smoke days increase perceptions of present and future threat, which is consistent with some previous studies (Berlin Rubin and Wong-Parodi, 2022; Buchanan et al., 2024; Duan and Bombara, 2023; McGee and Healey, 2022). Taken together, our findings and those from prior research suggest that people are not becoming complacent or habituated to wildfire smoke as concentrations increase and thus may continue to perceive high levels of risk and take protective actions even as community exposures become more frequent and severe.

Based on results from the full model (Fig. 5), a 1 *SD* increase in average PM2.5 on smoke days (about 4 μg/m^3^) predicted a modest 0.10 increase in the number of smoke-safe actions adopted, whereas subjective past smoke exposure was associated with adopting, on average, 1.11 more smoke-safe actions—indicating that parents who perceived prior exposure to wildfire smoke took more than one additional smoke-safe action like using an air purifier compared to parents who did not. Parents’ worry about wildfire smoke and their perceived susceptibility to future wildfire smoke events also consistently served as strong predictors of the number of smoke-safe actions taken (See Table S7). Although shared method variance may partly explain stronger associations between survey-based measures like perceived exposure and self-reported action adoption, our study suggests that subjective exposure reporting can still provide meaningful insights into wildfire smoke exposure; the results also build on existing literature suggesting that perceived exposure to wildfire smoke can motivate exposure-mitigating behaviours (Batdorf and McGee, 2023; Hano et al., 2020; Sugerman et al., 2012). This underscores the importance of subjective measures in capturing the psychological and behavioural responses to environmental threats, which likely guide actions more than objective air quality data alone. Furthermore, subjective measures of smoke based on environmental cues are crucial for guiding smoke-safe decision making in communities without nearby air monitors.

Several other studies have also explored how the adoption of protective measures against wildfire smoke differ by sociodemographic characteristics, though most studies have only linked this outcome with data from subjective smoke exposure measures. For example, similar to our results, Hano et al., (Hano et al., 2020) found that those who reported exposure to wildfire smoke in their community—and took protective behaviours like using air purifiers—were predominantly middle-age, female, and more highly educated. Other studies have similarly found that women are more likely than men to adopt smoke-safe actions and have attributed this to gendered differences in risk perception and perceived vulnerability to harm from wildfire smoke (Duan and Bombara, 2023; McGee and Healey, 2022). In addition, we observed an effect of family income on actions taken consistent with prior research. For example, Castillo et al. (2024) found that lower income individuals were significantly less likely to implement measures like replacing air filters, viewing these actions as too costly. However, any differences across income levels should not be interpreted as a lack of concern about wildfire smoke among low-income individuals. Rather, the differences observed can be explained by the constraints that lower incomes impose on available choices and behaviours, including reduced flexibility to stay indoors at home during smoke events (Burke et al., 2022). This highlights the socioeconomic barriers to adopting smoke-protective measures and underscores the need for more targeted programs and policies to close the gap and ensure more equitable access to cleaner air.

Some research has also shown that individuals with health conditions tend to take more precautions than those without. For example, MacIntyre et al. (2021) found that those with respiratory conditions were about twice as likely to report wearing a respirator during the 2019/2020 Australian Bushfire Season as those without. In our study, participants’ health conditions (and/or their children’s health conditions) had a significant effect on the number of actions taken. These results suggest that parents’ experiences and worry about smoke were strong predictors of protective behaviours, in line with findings by Hano et al. (2020), which demonstrated that many healthy individuals were proactive in adopting exposure-reducing actions due to high self-efficacy and a strong belief that wildfire smoke adversely affects health. Other studies have similarly found that parents tend to have higher smoke risk perceptions than adults without children (Buchanan et al., 2024; Santana et al., 2021), suggesting that even among parents with healthy children or who are themselves healthy, concern about future health impacts drives protective actions. As a result, concern appears to outweigh the influence of current health status, indicating that the perceived vulnerability of their families to wildfire smoke may be a more critical factor in motivating smoke-safe behaviours. This is encouraging, given that we know healthy children and adults can also experience health impacts from wildfire smoke exposure (Henderson et al., 2011; Rice et al., 2021), underscoring the importance of proactive exposure-mitigating actions regardless of current health status.

Similarly to Burke et al. (2022), we also observed evidence of non-linear relationships between some of our objective smoke exposure measures and the number of actions taken (Fig. 6). Specifically, our analysis revealed that, while initial increases in smoke exposure prompted more protective actions, this response eventually plateaued at more moderate levels of wildfire smoke before increasing again at higher smoke concentrations. This pattern implies that people may temporarily attenuate their behavioural response at certain levels of smoke exposure, but they appear to take more protective actions if exposure intensifies. Understanding this dynamic has important implications for public health practitioners, as it highlights the need for sustained and targeted public education to promote protective behaviours, even when individuals might initially feel that they have done enough to cope with smoke. Interestingly, the pattern we observed somewhat resembles the nonlinear relationship between PM2.5 concentrations and acute respiratory outcomes (Henderson et al., 2024), suggesting behavioural responses may be influenced by people experiencing acute health effects from smoke and by those who wish to prevent adverse health effects. However, the large confidence intervals and small number of data points at the highest level of wildfire smoke exposure suggest that more research is needed to further investigate these relationships.

This research has other important limitations, most notably, its reliance on a binary survey variable to measure participants’ subjective perceptions of their past smoke exposure. This measure likely over-simplifies participants’ experiences with wildfire smoke, potentially masking variations in the intensity or duration of their perceived exposure and limits our ability to capture more nuanced perspectives around perceptions of smoke exposure. This research also relied on participants’ recall of past wildfire seasons, which may not accurately reflect levels of smoke that occurred. Our dependent measure of the number of smoke-safe actions taken also asked participants to recall actions they had carried out in past wildfire seasons. Since a specific time period was not referenced in these survey items, we retrieved data on wildfire smoke measures from 2020 until survey administration date (Summer 2023) to generate our objective exposure measures; however, it’s possible participants’ actions and perceptions changed during the course of that period and likely continue to update over time. In future studies, administering a wildfire smoke log or diary for participants to track actions taken and their perceived exposure to smoke could provide a much more detailed and dynamic understanding of how protective behaviours evolve over time and how they correlate with subjective and objective wildfire smoke exposure.

Additionally, our objective exposure measures, which came from satellite-derived smoke plume data and PM2.5 observations from air quality monitors, have limitations such as the possibility of some non-differential exposure misclassification (Larsen et al., 2018; Schweizer et al., 2016). Nonetheless, using multiple measures of objective smoke exposure was a strength of this study, as it allowed us to examine which ones might be more influential in shaping people’s beliefs and behaviours related to smoke exposure. Our exploratory analysis, which investigated non-linear trends between these objective exposure measures and the number of actions taken, provided valuable insights into the complex relationship between varying levels of smoke exposure and protective behaviours, highlighting potential thresholds where action may increase or change. Lastly, although we used distance to the nearest air quality monitor to control for possible differences in the accuracy of urban/rural smoke measurements, our sample of participants was more urban than rural, and thus, our analysis likely underrepresented objective and subjective smoke exposure in more rural areas of the western US and Canada. Future research on wildfire smoke should prioritize surveying residents of rural areas, as these regions typically experience higher levels of smoke exposure and community members there may offer valuable insights on exposure-mitigating behaviours and experiences with wildfire smoke. Future work should also consider incorporating more individual-level exposure data (e.g., via biomonitoring, personal sampling) to allow for more precise estimates of objective wildfire smoke exposure, as this study relied on ambient PM2.5 concentrations at the residential postal/ZIP code level, which do not account for individual behaviors, mobility, or indoor air quality conditions.

## 5. Conclusions

In conclusion, this study offers important insights about which factors influence people to act to protect themselves and their families from wildfire smoke. Our results suggest that objective smoke exposure, perceptions of smoke exposure, and individual characteristics (e.g., health status) all play a role in motivating smoke-safe action adoption. Yet, subjective wildfire smoke exposure—smoke that parents perceive through sight or smell—appears to play an outsized role in this decision-making process. This finding is significant since most government risk communications aimed at guiding parents’ actions on wildfire smoke currently emphasize objective exposure measures (e.g., the AQI), and focus less on how parents perceive the threat of smoke and whether these perceptions are calibrated with objective risk of exposure.

To address this potential gap between subjective and objective smoke exposure, public health messaging could reference sensory cues as well as non-sensory cues, such as air quality indices, to help parents understand that even low levels of wildfire smoke, which may not be visible or detectable by smell, can pose a danger to health and warrant action. For example, messaging like “Even if you don’t smell smoke, the AQI is still moderate due to wildfire smoke—sensitive groups, like children, may be at risk” could bridge the gap between subjective and objective measures of smoke exposure and better align parents’ perceptions with objective air quality data. As wildfire smoke seasons grow longer, more frequent, and more intense across the western US and Canada and elsewhere, identifying effective strategies to encourage smoke protection and preparedness will be increasingly crucial for safeguarding public health.

## Supplementary Material

Supplementary Materials

Supplementary material associated with this article can be found, in the online version, at doi:10.1016/j.envc.2025.101362.

## Figures and Tables

**Table 1:** Standardized coefficients from regression models predicting the number of smoke-safe actions taken. Coefficients derived from objective smoke exposure (Model 1), subjective smoke exposure (Model 2), and the combination of objective and subjective exposure (Model 3). Objective indicators A through G differ by the objective measure(s) used as predictors. See Tables S4 and S5 for complete unstandardized and standardized regression coefficients for each model. There is only one subjective smoke exposure measure, thus, the coefficient for subjective smoke exposure is identical in Model Set 2 for each objective indicator used.

Objective Indicator	Predictors	Model Set 1 (Objective Only)	Model Set 2 (Subjective Only)	Model Set 3 (Objective + Subjective)
A	Total Number of Smoke days	**0.15 (0.07, 0.23)**		0.05 (−0.03, 0.12)
	Subjective Smoke Exposure		**1.71 (1.51, 1.90)**	**1.69 (1.49, 1.88)**
B	Total Number of Light Smoke Days	0.005 (−0.10, 0.11)		−0.004 (−0.10, 0.09)
	Total Number of Medium Smoke Days	−0.003 (−0.13, 0.12)		−0.03 (−0.15, 0.08)
	Total Number of Heavy Smoke Days	**0.16 (0.05, 0.27)**		0.08 (−0.02, 0.19)
	Subjective Smoke Exposure		**1.71 (1.51, 1.90)**	**1.68 (1.48, 1.88)**
C	Total Number of Smoke Days of PM2.5 > 15 μg/m^3^	**0.18 (0.08, 0.27)**		0.07 (−0.02, 0.16)
	Subjective Smoke Exposure		**1.71 (1.51, 1.90)**	**1.68 (1.49, 1.88)**
D	2-day Smoke Waves of PM2.5 > 15 μg/m^3^	**0.16 (0.08, 0.25)**		0.07 (−0.01, 0.15)
	Subjective Smoke Exposure		**1.71 (1.51, 1.90)**	**1.68 (1.49, 1.88)**
E	3-day Smoke Waves of PM2.5 > 15 μg/m^3^	**0.18 (0.10, 0.27)**		0.08 (−0.01, 0.16)
	Subjective Smoke Exposure		**1.71 (1.51, 1.90)**	**1.68 (1.48, 1.88)**
F	Number of Smoke Days With AQI > 100	**0.11 (0.03, 0.18)**		0.03 (−0.04, 0.10)
	Subjective Smoke Exposure		**1.71 (1.51, 1.90)**	**1.70 (1.50, 1.89)**
G	Average PM2.5 on Smoke Days	**0.21 (0.12, 0.29)**		**0.11 (0.04, 0.19)**
	Subjective Smoke Exposure		**1.71 (1.51, 1.90)**	**1.66 (1.47, 1.86)**

## Data Availability

Online repository containing all data and study materials is accessible via: https://osf.io/sytgp/.
