# Supplementary material for: Association between wildfire smoke exposure and parents’ adoption of protective behaviours: Exploring the role of objective and subjective smoke exposure
Source: Environ Chall (Amst). Author manuscript; Available in PMC 2026 Aug 6. (PMC13441431; doi:10.1016/j.envc.2025.101362)
Supplement: Supplementary file 1 [file NIHMS2191571-supplement-Supplementary_Materials.docx]

Online Supplementary Materials

**Association between wildfire smoke exposure and parents’ adoption of protective behaviours: Exploring the role of objective and subjective smoke exposure**

Catherine E. Slavik, Daniel A. Chapman, Stephanie E. Cleland, Perry Hystad, Ellen Peters

*Corresponding author. Email: [cslavik@uoregon.edu](mailto:cslavik@uoregon.edu).

**This PDF file includes:**

Tables S1 to S15

Supplementary Text

Figs. S1 to S5

**Table S1.** Demographic characteristics broken down by objective and subjective smoke exposure.

| Demographics | Level | Average PM2.5 on Smoke Days (quartiles in µg/m^3^) | | | | Subjective Past Smoke Exposure | |
| --- | --- | --- | --- | --- | --- | --- | --- |
|  |  | 0-25%  (4.34-12.75) | 25-50%  (12.75-15.50) | 50-75% (15.50-18.29) | 75-100% (18.29-36.22) | Yes | No |
| Gender | Man (%) | 42 | 47 | 46 | 31 | 41 | 48 |
|  | Woman (%) | 56 | 52 | 53 | 67 | 58 | 51 |
|  | Other (%) | 1 | 1 | 1 | 2 | 1 | 1 |
| Education | No Bachelor’s (%) | 42 | 54 | 53 | 68 | 47 | 53 |
|  | Bachelors or greater (%) | 58 | 46 | 47 | 32 | 45 | 55 |
| Health Condition | No (%) | 48 | 42 | 41 | 32 | 44 | 56 |
|  | Yes (%) | 52 | 58 | 59 | 68 | 62 | 38 |
| Jurisdiction | British Columbia (%) | 71 | 5 | 3 | 15 | 23 | 26 |
|  | California (%) | 13 | 41 | 30 | 20 | 24 | 37 |
|  | Oregon (%) | 7 | 9 | 33 | 51 | 27 | 15 |
|  | Washington (%) | 10 | 45 | 35 | 14 | 26 | 22 |
| Income | Median Income Bracket | 90-99,999 | 90-99,999 | 80-89,999 | 50-59,999 | 90-99,999 | 80-89,999 |
| Age | Median Age | 40 | 38 | 38 | 39 | 39 | 38 |

**Table S2.** Standardized parameter estimates for models predicting subjective exposure and perceived susceptibility from each objective smoke exposure measure (z-scored).

| **Model** | **Parameter** | **Subjective Exposure** | **Perceived Susceptibility** | **Worry** |
| --- | --- | --- | --- | --- |
|  |  | Odds Ratio  (95% CI) | Odds Ratio  (95% CI) | b (95% CI) |
| Model A | Intercept | 8.22 (6.27, 10.77) | 3.75 (3.27, 4.30) | 4.64 (4.57, 4.71) |
|  | Total number of smoke days | **1.88 (1.57, 2.26)** | **1.20 (1.06, 1.35)** | **0.12 (0.04, 0.20)** |
|  | Average PM2.5 on non-smoke days | 0.93 (0.81, 1.06) | 1.11 (0.99, 1.24) | **0.17 (0.10, 0.25)** |
|  | Distance to nearest monitoring station (log km) | 1.08 (0.93, 1.25) | 1.03 (0.91, 1.15) | 0.002 (-0.07, 0.08) |
| Model B | Intercept | 8.33 (6.35, 10.93) | 3.75 (3.27, 4.30) | 4.64 (4.57, 4.71) |
|  | Total number of light smoke days | 1.11 (0.91, 1.36) | 1.03 (0.89, 1.20) | -0.05 (-0.15, 0.04) |
|  | Total number of medium smoke days | 1.17 (0.91, 1.51) | 1.01 (0.84, 1.22) | 0.04 (-0.07, 0.16) |
|  | Total number of heavy smoke days | **1.63 (1.27, 2.08)** | **1.18 (1.00, 1.41)** | **0.12 (0.01, 0.22)** |
|  | Average PM2.5 on non-smoke days | 0.85 (0.71, 1.01) | 1.07 (0.92, 1.23) | **0.11 (0.02, 0.20)** |
|  | Distance to nearest monitoring station (log km) | 1.08 (0.93, 1.25) | 1.02 (0.91, 1.15) | -0.001 (-0.08, 0.07) |
| Model C | Intercept | 8.56 (6.45, 11.36) | 3.73 (3.26, 4.27) | 4.64 (4.57, 4.71) |
|  | Total number of smoke days exceeding 15 µg/m^3^ | **1.80 (1.47, 2.21)** | **1.29 (1.11, 1.50)** | **0.21 (0.11, 0.30)** |
|  | Average PM2.5 on non-smoke days | 0.57 (0.47, 0.68) | 0.91 (0.80, 1.05) | 0.02 (-0.07, 0.11) |
|  | Distance to nearest monitoring station (log km) | 1.01 (0.87, 1.17) | 1.01 (0.90, 1.13) | -0.003 (-0.08, 0.07) |
| Model D | Intercept | 8.71 (6.54, 11.62) | 3.74 (3.27, 4.28) | 4.64 (4.57, 4.71) |
|  | Two-day smoke waves where PM2.5 exceeded 15 µg/m^3^ | **1.74 (1.44, 2.10)** | **1.31 (1.14, 1.49)** | **0.12 (0.04, 0.21)** |
|  | Average PM2.5 on non-smoke days | 0.61 (0.51, 0.71) | 0.93 (0.82, 1.06) | **0.08 (0.00, 0.17)** |
|  | Distance to nearest monitoring station (log km) | 1.01 (0.87, 1.18) | 1.02 (0.91, 1.14) | -0.01 (-0.08, 0.06) |
| Model E | Intercept | 8.68 (6.52, 11.55) | 3.76 (3.28, 4.30) | 4.64 (4.57, 4.71) |
|  | Three-day smoke waves where PM2.5 exceeded 15 µg/m^3^ | **1.77 (1.47, 2.14)** | **1.29 (1.13, 1.48)** | **0.17 (0.09, 0.26)** |
|  | Average PM2.5 on non-smoke days | 0.60 (0.51, 0.70) | 0.93 (0.82, 1.06) | 0.05 (-0.04, 0.14) |
|  | Distance to nearest monitoring station (log km) | 1.00 (0.86, 1.16) | 1.01 (0.90, 1.13) | -0.01 (-0.08, 0.06) |
| Model F | Intercept | 8.73 (6.56, 11.60) | 3.74 (3.27, 4.28) | 4.64 (4.57, 4.71) |
|  | Number of smoke days where AQI reached “Unhealthy for Sensitive Groups” | **1.60 (1.33, 1.91)** | **1.18 (1.04, 1.33)** | **0.12 (0.04, 0.19)** |
|  | Average PM2.5 on non-smoke days | 0.76 (0.66, 0.87) | 1.04 (0.93, 1.17) | **0.13 (0.05, 0.20)** |
|  | Distance to nearest monitoring station (log km) | 1.00 (0.86, 1.16) | 1.00 (0.89, 1.12) | -0.01 (-0.09, 0.06) |
| Model G | Intercept | 8.43 (6.39, 11.12) | 3.76 (3.28, 4.31) | 4.64 (4.57, 4.72) |
|  | Average PM2.5 on smoke days | **1.76 (1.47, 2.11)** | **1.32 (1.16, 1.50)** | **0.16 (0.08, 0.24)** |
|  | Average PM2.5 on non-smoke days | 0.67 (0.58, 0.77) | 0.97 (0.86, 1.09) | **0.09 (0.01, 0.17)** |
|  | Distance to nearest monitoring station (log km) | 1.06 (0.90, 1.22) | 1.04 (0.93, 1.16) | 0.005 (-0.07, 0.08) |

*Note.* Each predictor was z-scored (M = 0, SD = 1) in each model. For subjective exposure, the random intercepts parameters (in SDs) were as follows: Model A = 0.65; Model B = 0.68; Model C = 0.86; Model D = 0.87; Model E = 0.87; Model F = 0.90; Model G = 0.76. For perceived susceptibility, the random intercepts parameters (in SDs) were as follows: Model A = 0.45; Model B = 0.45; Model C = 0.43; Model D = 0.41; Model E = 0.45; Model F = 0.46; Model G = 0.41. For worry, the random intercepts parameters (in SDs) were as follows: Model A = 0.36; Model B = 0.35; Model C = 0.34; Model D = 0.36; Model E = 0.35; Model F = 0.36; Model G = 0.35. Bolding indicates statistically significant results, with a p-value threshold of <0.05.

**Table S3.** Unstandardized parameter estimates for models predicting subjective exposure and perceived susceptibility from each objective smoke measure.

| **Model** | **Parameter** | **Subjective Exposure** | **Perceived Susceptibility** | **Worry** |
| --- | --- | --- | --- | --- |
|  |  | Odds Ratio (95% CI) | Odds Ratio (95% CI) | b  (95% CI) |
| Model A | Intercept | 0.20  (0.05, 0.73) | 0.88  (0.34, 2.23) | 3.39  (2.79, 3.99) |
|  | Total number of smoke days (in weeks) | **1.14  (1.10, 1.18)** | **1.04**  **(1.01, 1.06)** | **0.02**  **(0.01, 0.04)** |
|  | Average PM2.5 on non-smoke days (in 10 µg/m^3^) | 0.71  (0.38, 1.33) | 1.62  (0.95, 2.76) | **0.80**  **(0.45, 1.15)** |
|  | Distance to nearest monitoring station (log km) | 1.09  (0.92, 1.28) | 1.03  (0.91, 1.17) | 0.002  (-0.08, 0.08) |
| Model B | Intercept | 0.95  (0.11, 8.38) | 1.49  (0.30, 7.39) | 4.37  (3.33, 5.42) |
|  | Total number of light smoke days (in weeks) | 1.06 (0.95, 1.17) | 1.02  (0.94, 1.10) | -0.03  (-0.08, 0.02) |
|  | Total number of medium smoke days (in weeks) | 1.10 (0.95, 1.29) | 1.01  (0.90, 1.13) | 0.03  (-0.05, 0.10) |
|  | Total number of heavy smoke days (in weeks) | **1.19  (1.09, 1.30)** | **1.06**  **(1.00, 1.13)** | **0.04**  **(0.00, 0.08)** |
|  | Average PM2.5 on non-smoke days (in 10 µg/m^3^) | 0.46  (0.21, 1.04) | 1.34  (0.69, 2.61) | **0.52**  **(0.08, 0.96)** |
|  | Distance to nearest monitoring station (log km) | 1.08  (0.92, 1.27) | 1.03  (0.91, 1.16) | -0.001  (-0.08, 0.08) |
| Model C | Intercept | 17.00  (9.57, 30.19) | 3.03  (2.05, 4.50) | 4.20  (3.95, 4.46) |
|  | Total number of smoke days exceeding 15 µg/m^3^ (in weeks) | **1.18  (1.11, 1.25)** | **1.07**  **(1.03, 1.12)** | **0.06**  **(0.03, 0.08)** |
|  | Average PM2.5 on non-smoke days (in 10 µg/m^3^) | 0.07  (0.03, 0.16) | 0.66  (0.34, 1.27) | 0.08  (-0.35, 0.52) |
|  | Distance to nearest monitoring station (log km) | 1.01  (0.86, 1.19) | 1.01  (0.90, 1.14) | -0.004  (-0.08, 0.08) |
| Model D | Intercept | 12.47  (7.02, 22.14) | 2.58  (1.73, 3.85) | 4.13  (3.86, 4.39) |
|  | Two-day smoke waves where PM2.5 exceeded 15 µg/m^3^ (in waves) | **1.15  (1.10, 1.21)** | **1.07**  **(1.03, 1.11)** | **0.03**  **(0.01, 0.05)** |
|  | Average PM2.5 on non-smoke days (in 10 µg/m^3^) | 0.10  (0.04, 0.21) | 0.71  (0.39, 1.29) | 0.38  (-0.01, 0.78) |
|  | Distance to nearest monitoring station (log km) | 1.01  (0.86, 1.19) | 1.02  (0.90, 1.15) | -0.01  (-0.09, 0.07) |
| Model E | Intercept | 15.19  (8.59, 26.86) | 2.89  (1.95, 4.29) | 4.17  (3.91, 4.43) |
|  | Three-day smoke waves where PM2.5 exceeded 15 µg/m^3^ (in waves) | **1.21 (1.14, 1.29)** | **1.09**  **(1.04, 1.14)** | **0.06**  **(0.03, 0.09)** |
|  | Average PM2.5 on non-smoke days (in 10 µg/m^3^) | 0.09  (0.04, 0.19) | 0.71  (0.39, 1.31) | 0.24  (-0.17, 0.64) |
|  | Distance to nearest monitoring station (log km) | 1.00  (0.85, 1.18) | 1.01  (0.89, 1.14) | -0.01  (-0.09, 0.07) |
| Model F | Intercept | 10.41  (5.69, 19.04) | 2.58  (1.69, 3.93) | 4.08  (3.81, 4.35) |
|  | Number of smoke days where AQI reached “Unhealthy for Sensitive Groups” (in weeks) | **1.36 (1.21, 1.52)** | **1.11**  **(1.03, 1.20)** | **0.08**  **(0.03, 0.13)** |
|  | Average PM2.5 on non-smoke days (in 10 µg/m^3^) | 0.27  (0.14, 0.52) | 1.21  (0.72, 2.05) | **0.59**  **(0.24, 0.93)** |
|  | Distance to nearest monitoring station (log km) | 1.00  (0.85, 1.18) | 1.00  (0.89, 1.13) | -0.01  (-0.09, 0.07) |
| Model G | Intercept | 3.17  (1.53, 6.57) | 1.34  (0.78, 2.31) | 3.73  (3.38, 4.08) |
|  | Average PM2.5 on smoke days (in 10 µg/m^3^) | **3.89 (2.52, 6.00)** | **1.95**  **(1.43, 2.66)** | **0.39**  **(0.20, 0.58)** |
|  | Average PM2.5 on non-smoke days (in 10 µg/m^3^) | 0.15  (0.08, 0.30) | 0.87  (0.51, 1.50) | **0.42**  **(0.06, 0.78)** |
|  | Distance to nearest monitoring station (log km) | 1.05  (0.90, 1.24) | 1.04  (0.92, 1.18) | 0.006  (-0.08, 0.09) |

*Note.* For subjective exposure, the random intercepts parameters (in SDs) were as follows: Model A = 0.65; Model B = 0.68; Model C = 0.86; Model D = 0.87; Model E = 0.87; Model F = 0.90; Model G = 0.76. For perceived susceptibility, the random intercepts parameters (in SDs) were as follows: Model A = 0.45; Model B = 0.45; Model C = 0.43; Model D = 0.41; Model E = 0.45; Model F = 0.46; Model G = 0.41. For worry, the random intercepts parameters (in SDs) were as follows: Model A = 0.36; Model B = 0.35; Model C = 0.34; Model D = 0.36; Model E = 0.35; Model F = 0.36; Model G = 0.35. Bolding indicates statistically significant results, with a p-value threshold of <0.05.

**Table S4.** Full regression models summaries (standardized coefficients) predicting actions taken from objective smoke exposure (Model 1), subjective smoke exposure (Model 2), and the combination of objective and subjective exposure (Model 3) (z-scored).

| Model | Predictors | Model Set 1  (Objective Only) | Model Set 2 (Subjective Only) | Model Set 3  (Objective + Subjective) |
| --- | --- | --- | --- | --- |
| Model A | Intercept | 4.34 (4.26, 4.41) | 2.86 (2.68, 3.04) | 2.88 (2.69, 3.07) |
|  | Total number of smoke days | **0.15 (0.07, 0.23)** |  | 0.05 (-0.03, 0.12) |
|  | Subjective smoke exposure |  | **1.71 (1.51, 1.90)** | **1.69 (1.49, 1.88)** |
|  | Average PM2.5 on non-smoke days | **0.11 (0.04, 0.19)** | **0.13 (0.07, 0.20)** | **0.14 (0.07, 0.22)** |
|  | Distance to nearest monitoring station (log km) | -0.04 (-0.12, 0.04) | -0.06 (-0.13, 0.01) | -0.05 (-0.12, 0.02) |
| Model B | Intercept | 4.33 (4.26, 4.41) | 2.86 (2.68, 3.04) | 2.88 (2.70, 3.07) |
|  | Total number of light smoke days | 0.005 (-0.10, 0.11) |  | -0.004 (-0.10, 0.09) |
|  | Total number of medium smoke days | -0.003 (-0.13, 0.12) |  | -0.03 (-0.15, 0.08) |
|  | Total number of heavy smoke days | **0.16 (0.05, 0.27)** |  | 0.08 (-0.02, 0.19) |
|  | Subjective smoke exposure |  | **1.71 (1.51, 1.90)** | **1.68 (1.48, 1.88)** |
|  | Average PM2.5 on non-smoke days | 0.06 (-0.04, 0.16) | **0.13 (0.07, 0.20)** | **0.11 (0.02, 0.20)** |
|  | Distance to nearest monitoring station (log km) | -0.04 (-0.12, 0.04) | -0.06 (-0.13, 0.01) | -0.05 (-0.12, 0.02) |
| Model C | Intercept | 4.33 (4.25, 4.41) | 2.86 (2.68, 3.04) | 2.88 (2.70, 3.06) |
|  | Total number of smoke days exceeding 15 µg/m^3^ | **0.18 (0.08, 0.27)** |  | 0.07 (-0.02, 0.16) |
|  | Subjective smoke exposure |  | **1.71 (1.51, 1.90)** | **1.68 (1.49, 1.88)** |
|  | Average PM2.5 on non-smoke days | -0.03 (-0.12, 0.07) | **0.13 (0.07, 0.20)** | **0.09 (0.00, 0.18)** |
|  | Distance to nearest monitoring station (log km) | -0.05 (-0.13, 0.02) | -0.06 (-0.13, 0.01) | -0.05 (-0.12, 0.02) |
| Model D | Intercept | 4.33 (4.26, 4.41) | 2.86 (2.68, 3.04) | 2.88 (2.70, 3.07) |
|  | Two-day smoke waves where PM2.5 exceeded 15 µg/m^3^ | **0.16 (0.08, 0.25)** |  | 0.07 (-0.01, 0.15) |
|  | Subjective smoke exposure |  | **1.71 (1.51, 1.90)** | **1.68 (1.49, 1.88)** |
|  | Average PM2.5 on non-smoke days | -0.002 (-0.09, 0.09) | **0.13 (0.07, 0.20)** | **0.10 (0.02, 0.18)** |
|  | Distance to nearest monitoring station (log km) | -0.05 (-0.13, 0.03) | -0.06 (-0.13, 0.01) | -0.05 (-0.12, 0.02) |
| Model E | Intercept | 4.33 (4.26, 4.41) | 2.86 (2.68, 3.04) | 2.89 (2.70, 3.07) |
|  | Three-day smoke waves where PM2.5 exceeded 15 µg/m^3^ | **0.18 (0.10, 0.27)** |  | 0.08 (-0.01, 0.16) |
|  | Subjective smoke exposure |  | **1.71 (1.51, 1.90)** | **1.68 (1.48, 1.88)** |
|  | Average PM2.5 on non-smoke days | -0.02 (-0.11, 0.07) | **0.13 (0.07, 0.20)** | **0.09 (0.01, 0.18)** |
|  | Distance to nearest monitoring station (log km) | -0.06 (-0.13, 0.02) | -0.06 (-0.13, 0.01) | -0.05 (-0.12, 0.02) |
| Model F | Intercept | 4.33 (4.25, 4.41) | 2.86 (2.68, 3.04) | 2.87 (2.69, 3.05) |
|  | Number of smoke days where AQI reached “Unhealthy for Sensitive Groups” | **0.11 (0.03, 0.18)** |  | 0.03 (-0.04, 0.10) |
|  | Subjective smoke exposure |  | **1.71 (1.51, 1.90)** | **1.70 (1.50, 1.89)** |
|  | Average PM2.5 on non-smoke days | 0.07 (-0.01, 0.14) | **0.13 (0.07, 0.20)** | **0.13 (0.06, 0.20)** |
|  | Distance to nearest monitoring station (log km) | -0.06 (-0.14, 0.02) | -0.06 (-0.13, 0.01) | -0.06 (-0.13, 0.01) |
| Model G | Intercept | 4.34 (4.27, 4.42) | 2.86 (2.68, 3.04) | 2.90 (2.72, 3.09) |
|  | Average PM2.5 on smoke days | **0.21 (0.12, 0.29)** |  | **0.11 (0.04, 0.19)** |
|  | Subjective smoke exposure |  | **1.71 (1.51, 1.90)** | **1.66 (1.47, 1.86)** |
|  | Average PM2.5 on non-smoke days | 0.01 (-0.07, 0.09) | **0.13 (0.07, 0.20)** | **0.09 (0.02, 0.17)** |
|  | Distance to nearest monitoring station (log km) | -0.03 (-0.11, 0.04) | -0.06 (-0.13, 0.01) | -0.04 (-0.11, 0.03) |

*Note.* Each continue measure was z-scored (M = 0, SD = 1) for analysis. Subjective smoke exposure is a binary coded variable and was not z-scored. The random intercepts parameters (in SDs) for Model 1’s were as follows: Model A = 0.48; Model B = 0.49; Model C = 0.49; Model D = 0.49; Model E = 0.49; Model F = 0.50; Model G = 0.44. The random intercepts parameter (in SD) for Model 2 A = 0.42. There is only one subjective smoke exposure measure, thus, the coefficient for subjective smoke exposure is identical through Models A-G in Model Set 2. The random intercepts parameters (in SDs) for Model 3’s were as follows: Model A = 0.43; Model B = 0.43; Model C = 0.43; Model D = 0.43; Model E = 0.43; Model F = 0.43; Model G = 0.40. Bolding indicates statistically significant results, with a p-value threshold of <0.05.

**Table S5.** Full regression models summaries (standardized coefficients) predicting actions taken from objective smoke exposure (Model 1), subjective smoke exposure (Model 2), and the combination of objective and subjective exposure (Model 3).

| **Model** | **Predictors** | **Model Set 1**  **(Objective Only)** | **Model Set 2 (Subjective Only)** | **Model Set 3**  **(Objective + Subjective)** |
| --- | --- | --- | --- | --- |
| Model A | Intercept | 3.12 (2.50, 3.74) | 2.51 (2.20, 2.82) | 2.20 (1.61, 2.79) |
|  | Total number of smoke days (in weeks) | **0.03 (0.01, 0.05)** |  | 0.01 (-0.01, 0.02) |
|  | Subjective smoke exposure |  | **1.71 (1.51, 1.90)** | **1.69 (1.49, 1.88)** |
|  | Average PM2.5 on non-smoke days (10 µg/m^3^ increments) | **0.53 (0.18, 0.89)** | **0.63 (0.30, 0.95)** | **0.67 (0.34, 1.01)** |
|  | Distance to nearest monitoring station (log km) | -0.04 (-0.13, 0.04) | -0.07 (-0.14, 0.01) | -0.05 (-0.13, 0.02) |
| Model B | Intercept | 3.85 (2.77, 4.92) | 2.51 (2.20, 2.82) | 2.61 (1.59, 3.63) |
|  | Total number of light smoke days (in weeks) | 0.003 (-0.05, 0.06) |  | -0.002 (-0.05, 0.05) |
|  | Total number of medium smoke days (in weeks) | -0.02 (-0.08, 0.07) |  | -0.02 (-0.09, 0.05) |
|  | Total number of heavy smoke days (in weeks) | **0.06 (0.02, 0.10)** |  | 0.03 (-0.01, 0.07) |
|  | Subjective smoke exposure |  | **1.71 (1.51, 1.90)** | **1.68 (1.48, 1.88)** |
|  | Average PM2.5 on non-smoke days (10 µg/m^3^ increments) | 0.28 (-0.17, 0.73) | **0.63 (0.30, 0.95)** | **0.51 (0.09, 0.94)** |
|  | Distance to nearest monitoring station (log km) | -0.04 (-0.13, 0.04) | -0.07 (-0.14, 0.01) | -0.06 (-0.14, 0.02) |
| Model C | Intercept | 4.15 (3.88, 4.42) | 2.51 (2.20, 2.82) | 2.53 (2.22, 2.84) |
|  | Total number of smoke days exceeding 15 µg/m^3^ (in weeks) | **0.05 (0.02, 0.08)** |  | 0.02 (-0.01, 0.04) |
|  | Subjective smoke exposure |  | **1.71 (1.51, 1.90)** | **1.68 (1.49, 1.88)** |
|  | Average PM2.5 on non-smoke days (10 µg/m^3^ increments) | -0.13 (-0.57, 0.23) | **0.63 (0.30, 0.95)** | **0.42 (0.00, 0.84)** |
|  | Distance to nearest monitoring station (log km) | -0.06 (-0.14, 0.03) | -0.07 (-0.14, 0.01) | -0.06 (0.14, 0.02) |
| Model D | Intercept | 4.05 (3.77, 4.32) | 2.51 (2.20, 2.82) | 2.49 (2.17, 2.80) |
|  | Two-day smoke waves where PM2.5 exceeded 15 µg/m^3^ | **0.04 (0.02, 0.07)** |  | 0.02 (0.00, 0.04) |
|  | Subjective smoke exposure |  | **1.71 (1.51, 1.90)** | **1.68 (1.49, 1.88)** |
|  | Average PM2.5 on non-smoke days (10 µg/m^3^ increments) | -0.01 (-0.42, -0.40) | **0.63 (0.30, 0.95)** | **0.46 (0.08, 0.84)** |
|  | Distance to nearest monitoring station (log km) | -0.06 (-0.14, 0.03) | -0.07 (-0.14, 0.01) | -0.06 (-0.14, 0.02) |
| Model E | Intercept | 4.11 (3.84, 4.38) | 2.51 (2.20, 2.82) | 2.52 (2.20, 2.83) |
|  | Three-day smoke waves where PM2.5 exceeded 15 µg/m^3^ | **0.06 (0.03, 0.09)** |  | 0.03 (0.00, 0.05) |
|  | Subjective smoke exposure |  | **1.71 (1.51, 1.90)** | **1.68 (1.48, 1.88)** |
|  | Average PM2.5 on non-smoke days (10 µg/m^3^ increments) | -0.09 (-0.50, 0.33) | **0.63 (0.30, 0.95)** | **0.42 (0.03, 0.81)** |
|  | Distance to nearest monitoring station (log km) | -0.06 (-0.14, 0.02) | -0.07 (-0.14, 0.01) | -0.06 (-0.14, 0.02) |
| Model F | Intercept | 4.04 (3.76, 4.32) | 2.51 (2.20, 2.82) | 2.49 (2.17, 2.81) |
|  | Number of smoke days where AQI reached “Unhealthy for Sensitive Groups” (in weeks) | **0.07 (0.02, 0.12)** |  | 0.02 (-0.03, 0.07) |
|  | Subjective smoke exposure |  | **1.71 (1.51, 1.90)** | **1.70 (1.50, 1.89)** |
|  | Average PM2.5 on non-smoke days (10 µg/m^3^ increments) | 0.30 (-0.05, 0.66) | **0.63 (0.30, 0.95)** | **0.60 (0.27, 0.93)** |
|  | Distance to nearest monitoring station (log km) | -0.06 (-0.15, 0.02) | -0.07 (-0.14, 0.01) | -0.06 (-0.14, 0.02) |
| Model G | Intercept | 3.56 (3.20, 3.91) | 2.51 (2.20, 2.82) | 2.22 (1.85, 2.59) |
|  | Average PM2.5 on smoke days (10 µg/m^3^ increments) | **0.49 (0.30, 0.69)** |  | **0.27 (0.09, 0.46)** |
|  | Subjective smoke exposure |  | **1.71 (1.51, 1.90)** | **1.66 (1.47, 1.86)** |
|  | Average PM2.5 on non-smoke days (10 µg/m^3^ increments) | 0.05 (-0.32, 0.41) | **0.63 (0.30, 0.95)** | **0.44 (0.09, 0.78)** |
|  | Distance to nearest monitoring station (log km) | -0.04 (-0.12, 0.05) | -0.07 (-0.14, 0.01) | -0.04 (-0.12, 0.03) |

*Note.* The random intercepts parameters (in SDs) for Model 1’s were as follows: Model A = 0.48; Model B = 0.49; Model C = 0.49; Model D = 0.49; Model E = 0.49; Model F = 0.50; Model G = 0.44. The random intercepts parameter (in SD) for Model 2 A = 0.42. There is only one subjective smoke exposure measure, thus, the coefficient for subjective smoke exposure is identical through Models A-G in Model Set 2. The random intercepts parameters (in SDs) for Model 3’s were as follows: Model A = 0.43; Model B = 0.43; Model C = 0.43; Model D = 0.43; Model E = 0.43; Model F = 0.43; Model G = 0.40. Bolding indicates statistically significant results, with a p-value threshold of <0.05.

**Table S6.** Model AIC Values Predicting Actions Taken from Each Subjective and Objective Smoke Predictor).

| **Model** | **AIC** |
| --- | --- |
| Subjective smoke exposure | 7462.60 |
| Total number of smoke days | 7727.52 |
| Total number of light smoke days | 7737.36 |
| Total number of medium smoke days |  |
| Total number of heavy smoke days |  |
| Total number of smoke days exceeding 15 µg/m^3^ | 7726.73 |
| Two-day smoke waves where PM2.5 exceeded 15 µg/m^3^ | 7726.61 |
| Three-day smoke waves where PM2.5 exceeded 15 µg/m^3^ | 7723.00 |
| Number of smoke days where AQI reached “Unhealthy for Sensitive Groups” | 7730.93 |
| Average PM2.5 on smoke days (10 µg/m^3^ increments) | 7711.40 |

*Note.* AIC = Akaike Information Criterion. Lower AIC values are preferred. Each row represents a separate hierarchical linear regression model, except for light, medium, and heavy smoke days, which were included in a single model together. Controls in each model included average PM2.5 on non-smoke days and the distance to the nearest monitoring station.

|  | **Model A** | **Model B** | **Model C** | **Model D** | **Model E** | **Model F** | **Model G** |
| --- | --- | --- | --- | --- | --- | --- | --- |
| Predictors | Total number of smoke days (in weeks) | Total number of light/  medium/  heavy smoke days (in weeks) | Total number of smoke days exceeding 15 µg/m^3^ (in weeks) | Two-day smoke waves where PM2.5 exceeded 15 µg/m^3^ | Three-day smoke waves where PM2.5 exceeded 15 µg/m^3^ | Number of smoke days where AQI reached “Unhealthy for Sensitive Groups” (in weeks) | Average PM2.5 on smoke days (10 µg/m^3^ increments) |
| Intercept | 2.63 (2.41, 2.85) | 2.63 (2.41, 2.85) | 2.62 (2.40, 2.84) | 2.63 (2.41, 2.85) | 2.63 (2.41, 2.85) | 2.61 (2.39, 2.83) | 2.65 (2.43, 2.87) |
| Objective Measure (z) | 0.06 (-0.01, 0.13) | 0.02 (-0.06, 0.10) /  -0.03 (-0.14, 0.07)  /  0.08 (-0.01, 0.17) | 0.05 (-0.03, 0.13) | 0.07 (-0.01, 0.14) | 0.06 (-0.02, 0.14) | 0.02 (-0.04, 0.09) | **0.10 (0.03, 0.17)** |
| Subjective Past Exposure | **1.12 (0.93, 1.31)** | **1.12 (0.93, 1.31)** | **1.13 (0.94, 1.32)** | **1.12 (0.93, 1.31)** | **1.12 (0.93, 1.31)** | **1.13 (0.94, 1.32)** | **1.11 (0.92, 1.30)** |
| Perceived Future Susceptibility | **0.53 (0.36, 0.69)** | **0.53 (0.36, 0.69)** | **0.53 (0.37, 0.69)** | **0.52 (0.36, 0.68)** | **0.53 (0.36, 0.69)** | **0.53 (0.37, 0.69)** | **0.52 (0.36, 0.68)** |
| Worry (z) | **0.48 (0.42, 0.55)** | **0.48 (0.42, 0.55)** | **0.48 (0.42, 0.55)** | **0.48 (0.42, 0.55)** | **0.48 (0.42, 0.55)** | **0.49 (0.42, 0.55)** | **0.48 (0.42, 0.55)** |
| Bachelor’s Degree or Greater | **0.16 (0.02, 0.30)** | **0.16 (0.02, 0.30)** | **0.15 (0.01, 0.29)** | **0.16 (0.02, 0.29)** | **0.15 (0.01, 0.29)** | **0.15 (0.01, 0.29)** | **0.16 (0.02, 0.30)** |
| Health Condition | **0.29 (0.17, 0.42)** | **0.29 (0.17, 0.42)** | **0.30 (0.17, 0.43)** | **0.30 (0.17, 0.43)** | **0.30 (0.17, 0.43)** | **0.30 (0.17, 0.42)** | **0.29 (0.17, 0.42)** |
| Family Income (z) | **0.15 (0.08, 0.22)** | **0.15 (0.08, 0.22)** | **0.14 (0.07, 0.22)** | **0.15 (0.07, 0.22)** | **0.14 (0.07, 0.22)** | **0.14 (0.07, 0.22)** | **0.15 (0.08, 0.23)** |
| Gender: Man vs. Other | -0.004 (-0.58, 0.57) | -0.02 (-0.59, 0.56) | -0.01 (-0.59, 0.56) | -0.01 (-0.59, 0.56) | -0.01 (-0.59, 0.56) | -0.01 (-0.59, 0.56) | -0.01 (-0.58, 0.57) |
| Gender: Man vs. Woman | **0.16 (0.03, 0.29)** | **0.16 (0.03, 0.29)** | **0.16 (0.03, 0.29)** | **0.16 (0.02, 0.29)** | **0.16 (0.03, 0.29)** | **0.16 (0.03, 0.29)** | **0.14 (0.01, 0.28)** |
| Age (z) | **-0.20 (-0.26, -0.14)** | **-0.20 (-0.26, -0.14)** | **-0.20 (-0.26, -0.14)** | **-0.20 (-0.26, -0.14)** | **-0.20 (-0.26, -0.14)** | **-0.20 (-0.26, -0.14)** | **-0.20 (-0.26, -0.14)** |
| Avg. PM2.5 on non-smoke days (z) | 0.07 (0.00, 0.14) | 0.05 (-0.03, 0.13) | 0.03 (-0.05, 0.11) | 0.02 (-0.05, 0.10) | 0.02 (-0.05, 0.10) | 0.05 (-0.01, 0.12) | 0.02 (-0.05, 0.09) |
| Distance to Monitoring Station (log) (z) | -0.06 (-0.12, 0.01) | -0.06 (-0.12, 0.01) | -0.06 (-0.13, 0.00) | -0.06 (-0.12, 0.00) | -0.06 (-0.13, 0.00) | -0.06 (-0.13, 0.00) | -0.05 (-0.11, 0.01) |

.

*Note.* The random intercepts parameters (in SDs) for Model 3’s were as follows: Model A = 0.30; Model B = 0.30; Model C = 0.30; Model D = 0.30; Model E = 0.30; Model F = 0.30; Model G = 0.27. Bolding indicates statistically significant results, with a p-value threshold of <0.05. Values marked with (z) denote z-scored variables (mean = 0, standard deviation = 1).

**Table S7.** Parameters Estimates for Model Set 4 containing standardized regression coefficients.

|  | **Model A** | **Model B** | **Model C** | **Model D** | **Model E** | **Model F** | **Model G** |
| --- | --- | --- | --- | --- | --- | --- | --- |
| Predictors | Total number of smoke days (in weeks) | Total number of light/  medium/  heavy smoke days (in weeks) | Total number of smoke days exceeding 15 µg/m^3^ (in weeks) | Two-day smoke waves where PM2.5 exceeded 15 µg/m^3^ | Three-day smoke waves where PM2.5 exceeded 15 µg/m^3^ | Number of smoke days where AQI reached “Unhealthy for Sensitive Groups” (in weeks) | Average PM2.5 on smoke days (10 µg/m^3^ increments) |
| Intercept | 2.14 (1.58, 2.71) | 2.35 (1.43, 3.28) | 2.52 (2.19, 2.84) | 2.48 (2.16, 2.81) | 2.51 (2.18, 2.84) | 2.49 (2.16, 2.82) | 2.27 (1.90, 2.64) |
| Objective Measure | 0.01 (0.00, 0.03) | 0.01 (-0.03, 0.05) /  -0.02 (-0.08, 0.04) /  0.03 (0.00, 0.06) | 0.01 (-0.01, 0.04) | 0.02 (0.00, 0.04) | 0.02 (-0.01, 0.05) | 0.02 (-0.03, 0.06) | **0.23 (0.06, 0.40)** |
| Subjective Past Exposure | **1.12 (0.93, 1.31)** | **1.12 (0.93, 1.31)** | **1.13 (0.94, 1.32)** | **1.12 (0.93, 1.31)** | **1.12 (0.93, 1.31)** | **1.13 (0.94, 1.32)** | **1.11 (0.92, 1.30)** |
| Perceived Future Susceptibility | **0.53 (0.36, 0.69)** | **0.53 (0.36, 0.69)** | **0.53 (0.37, 0.69)** | **0.52 (0.36, 0.68)** | **0.53 (0.36, 0.69)** | **0.53 (0.37, 0.69)** | **0.52 (0.36, 0.68)** |
| Worry (z) | **0.48 (0.42, 0.55)** | **0.48 (0.42, 0.55)** | **0.48 (0.42, 0.55)** | **0.48 (0.42, 0.55)** | **0.48 (0.42, 0.55)** | **0.49 (0.42, 0.55)** | **0.48 (0.42, 0.55)** |
| Bachelor’s Degree or Greater | **0.16 (0.02, 0.30)** | **0.16 (0.02, 0.30)** | **0.15 (0.01, 0.29)** | **0.16 (0.02, 0.29)** | **0.15 (0.01, 0.29)** | **0.15 (0.01, 0.29)** | **0.16 (0.02, 0.30)** |
| Health Condition | **0.29 (0.17, 0.42)** | **0.29 (0.17, 0.42)** | **0.30 (0.17, 0.43)** | **0.30 (0.17, 0.43)** | **0.30 (0.17, 0.43)** | **0.30 (0.17, 0.42)** | **0.29 (0.17, 0.42)** |
| Family Income (z) | **0.15 (0.08, 0.22)** | **0.15 (0.08, 0.22)** | **0.14 (0.07, 0.22)** | **0.15 (0.07, 0.22)** | **0.14 (0.07, 0.22)** | **0.14 (0.07, 0.22)** | **0.15 (0.08, 0.23)** |
| Gender: Man vs. Other | -0.004 (-0.58, 0.57) | -0.02 (-0.59, 0.56) | -0.01 (-0.59, 0.56) | -0.01 (-0.59, 0.56) | -0.01 (-0.59, 0.56) | -0.01 (-0.59, 0.56) | -0.01 (-0.58, 0.57) |
| Gender: Man vs. Woman | **0.16 (0.03, 0.29)** | **0.16 (0.03, 0.29)** | **0.16 (0.03, 0.29)** | **0.16 (0.02, 0.29)** | **0.16 (0.03, 0.29)** | **0.16 (0.03, 0.29)** | **0.14 (0.01, 0.28)** |
| Age (z) | **-0.20 (-0.26, -0.14)** | **-0.20 (-0.26, -0.14)** | **-0.20 (-0.26, -0.14)** | **-0.20 (-0.26, -0.14)** | **-0.20 (-0.26, -0.14)** | **-0.20 (-0.26, -0.14)** | **-0.20 (-0.26, -0.14)** |
| Avg. PM2.5 on non-smoke days | **0.33 (0.02, 0.63)** | 0.22 (-0.16, 0.60) | 0.14 (-0.25, 0.52) | 0.11 (-0.24, 0.46) | 0.12 (-0.24, 0.48) | 0.25 (-0.05, 0.56) | 0.10 (-0.22, 0.43) |
| Distance to Monitoring Station (log) | -0.06 (-0.13, 0.01) | -0.06 (-0.13, 0.01) | -0.07 (-0.14, 0.00) | -0.07 (-0.13, 0.00) | -0.07 (-0.14, 0.00) | -0.07 (-0.14, 0.00) | -0.06 (-0.13, 0.01) |

*Note.* The random intercepts parameters (in SDs) for Model 3’s were as follows: Model A = 0.30; Model B = 0.30; Model C = 0.30; Model D = 0.30; Model E = 0.30; Model F = 0.30; Model G = 0.27. Bolding indicates statistically significant results, with a p-value threshold of <0.05. Values marked with (z) denote z-scored variables (mean = 0, standard deviation = 1).

**Table S8.** Parameters Estimates for Model Set 4 containing unstandardized regression coefficients.

| **Model** |  | **F** | **Expected Degrees of Freedom** | **p-value** |
| --- | --- | --- | --- | --- |
| Model A | **Total number of smoke days (in weeks)** | **6.80** | **1.96** | **0.002** |
|  | **Average PM2.5 on non-smoke days (10 µg/m^3^ increments)** | **8.09** | **1.74** | **0.004** |
| Model B | Total number of light smoke days (in weeks) | 1.88 | 4.69 | 0.076 |
|  | Total number of medium smoke days (in weeks) | 0.47 | 2.30 | 0.497 |
|  | **Total number of heavy smoke days (in weeks)** | **4.63** | **3.83** | **0.001** |
|  | Average PM2.5 on non-smoke days (10 µg/m^3^ increments) | 3.24 | 1.00 | 0.072 |
| Model C | **Total number of smoke days exceeding 15 µg/m^3^ (in weeks)** | **9.30** | **3.95** | **< 0.001** |
|  | Average PM2.5 on non-smoke days (10 µg/m^3^ increments) | 0.82 | 1.00 | 0.365 |
| Model D | Two-day smoke waves where PM2.5 exceeded 15 µg/m^3^ | 0.65 | 1.00 | 0.420 |
|  | **Average PM2.5 on non-smoke days (10 µg/m^3^ increments)** | **3.51** | **3.51** | **0.004** |
| Model E | Three-day smoke waves where PM2.5 exceeded 15 µg/m^3^ | 1.79 | 1.00 | 0.181 |
|  | **Average PM2.5 on non-smoke days (10 µg/m^3^ increments)** | **4.30** | **3.54** | **0.007** |
| Model F | **Number of smoke days where AQI reached “Unhealthy for Sensitive Groups” (in weeks)** | **4.41** | **3.83** | **0.003** |
|  | **Average PM2.5 on non-smoke days (10 µg/m^3^ increments)** | **7.86** | **1.64** | **0.008** |
| Model G | **Average PM2.5 on smoke days (10 µg/m^3^ increments)** | **10.13** | **3.61** | **< 0.001** |
|  | Average PM2.5 on non-smoke days (10 µg/m^3^ increments) | 0.01 | 1.00 | 0.910 |

**Table S9.** Smooth Terms from Non-Linear Multilevel Regression Model Using Cubic Regression Splines.

**Table S10.**Linear (Non-Smooth) Terms from Non-Linear Multilevel Regression Model Using Cubic Regression Splines.

| **Model** | **Linear Term** | **b (SE)** | **p-value** |
| --- | --- | --- | --- |
| Model A | Intercept | 4.36 (0.06) | < 0.001 |
|  | Distance to nearest monitoring station (log km) | -0.04 (0.04) | 0.361 |
| Model B | Intercept | 4.35 (0.06) | < 0.001 |
|  | Distance to nearest monitoring station (log km) | -0.03 (0.04) | 0.505 |
| Model C | Intercept | 4.37 (0.06) | < 0.001 |
|  | Distance to nearest monitoring station (log km) | -0.03 (0.04) | 0.387 |
| Model D | Intercept | 4.38 (0.06) | < 0.001 |
|  | Distance to nearest monitoring station (log km) | -0.06 (0.04) | 0.160 |
| Model E | Intercept | 4.38 (0.06) | < 0.001 |
|  | Distance to nearest monitoring station (log km) | -0.06 (0.04) | 0.162 |
| Model F | Intercept | 4.38 (0.06) | < 0.001 |
|  | Distance to nearest monitoring station (log km) | -0.05 (0.04) | 0.188 |
| Model G | Intercept | 4.36 (0.06) | < 0.001 |
|  | Distance to nearest monitoring station (log km) | -0.02 (0.04) | 0.552 |

Supplementary Text

Exploratory Non-linear Moderation Analyses

For the exploratory non-linear moderation analyses, we elected to use average PM2.5 on smoke days as our focal indicator of smoke exposure, given its robustness as a predictor for actions taken (relative to the other smoke exposure measures). For demographics, we examine each demographic included in our ensemble of predictors, i.e., education, age, gender, income, and the presence of health conditions. For gender, we omitted the ‘other’ category as there were fewer than 30 participants in that category and we did not feel that was sufficient to properly estimate the nonlinear slope. For income, we created a three-level variable by splitting the income brackets into bins of < $50,000, $50-100,000, and > $100,000. For age, we binned the continuous measure into three levels: 18-34, 35-54, and 55 and over. In each model, we included the same two controls as all other models and included random intercepts for postal/zip code. Figures S1-S5 display the results of the models graphically, and Tables S11-S15 provide the parameter estimates. For the parametric terms, we report unstandardized regression coefficients (b), standard errors (SE), and p-values. For the non-parametric smoothed terms, we report F values, estimated degrees of freedom (EDF), and p-values.

**Table S11.** Parameter Estimates from Model with Education Levels as Separate Splines.

| **Coefficient Type** |  | **b** | **SE** | **p-value** |
| --- | --- | --- | --- | --- |
| Parametric | Intercept | 4.19 | 0.07 | < 0.001 |
|  | **Education (ref = Less than Bachelor’s)** | **0.38** | **0.07** | **< 0.001** |
|  | Distance to nearest monitoring station (log) | -0.03 | 0.04 | 0.425 |
|  |  | F | EDF | p-value |
| Non-parametric smooth | **Avg. PM2.5 on smoke days: Less than Bachelor’s** | **16.59** | **1.70** | **< 0.001** |
|  | **Avg. PM2.5 on smoke days: Bachelor’s or greater** | **8.65** | **3.64** | **< 0.001** |
|  | Avg. PM2.5 on non-smoke days | 0.33 | 1 | 0.562 |

**Table S12.** Parameter Estimates from Model with Gender Levels as Separate Splines.

| **Coefficient Type** |  | **b** | **SE** | **p-value** |
| --- | --- | --- | --- | --- |
| Parametric | Intercept | 4.24 | 0.07 | < 0.001 |
|  | **Gender (ref = Men)** | **0.22** | **0.08** | **0.005** |
|  | Distance to nearest monitoring station (log) | -0.04 | 0.04 | 0.367 |
|  |  | F | EDF | p-value |
| Non-parametric smooth | **Avg. PM2.5 on smoke days: Men** | **4.70** | **3.68** | **0.001** |
|  | **Avg. PM2.5 on smoke days: Women** | **7.87** | **2.31** | **< 0.001** |
|  | Avg. PM2.5 on non-smoke days | 0.56 | 1 | 0.453 |

| **Coefficient Type** |  | **b** | **SE** | **p-value** |
| --- | --- | --- | --- | --- |
| Parametric | Intercept | 4.13 | 0.08 | < 0.001 |
|  | **Income: 50-100,000 (ref = < 50,000)** | **0.21** | **0.09** | **0.027** |
|  | **Income: Over 100,000 (ref = < 50,000)** | **0.47** | **0.09** | **< 0.001** |
|  | Distance to nearest monitoring station (log) | -0.03 | 0.04 | 0.437 |
|  |  | F | EDF | p-value |
| Non-parametric smooth | **Avg. PM2.5 on smoke days: < 50,000** | **10.58** | **1.34** | **0.001** |
|  | **Avg. PM2.5 on smoke days: 50-100,000** | **5.78** | **4.19** | **< 0.001** |
|  | **Avg. PM2.5 on smoke days: > 100,000** | **7.37** | **3.31** | **< 0.001** |
|  | Avg. PM2.5 on non-smoke days | 1.45 | 1 | 0.229 |

**Table S13.** Parameter Estimates from Model with Income Levels as Separate Splines.

**Table S14.** Parameter Estimates from Model with Health Condition Levels as Separate Splines.

| **Coefficient Type** |  | **b** | **SE** | **p-value** |
| --- | --- | --- | --- | --- |
| Parametric | Intercept | 4.04 | 0.07 | < 0.001 |
|  | **Health condition (ref = No)** | **0.53** | **0.07** | **< 0.001** |
|  | Distance to nearest monitoring station (log) | -0.02 | 0.04 | 0.633 |
|  |  | F | EDF | p-value |
| Non-parametric smooth | **Avg. PM2.5 on smoke days: No health conditions** | **6.84** | **3.59** | **< 0.001** |
|  | **Avg. PM2.5 on smoke days: Yes health conditions** | **14.82** | **1** | **< 0.001** |
|  | Avg. PM2.5 on non-smoke days | 0.11 | 1 | 0.743 |

**Table S15.** Parameter Estimates from Model with Age Levels as Separate Splines.

| **Coefficient Type** |  | **b** | **SE** | **p-value** |
| --- | --- | --- | --- | --- |
| Parametric | Intercept | 4.44 | 0.08 | < 0.001 |
|  | Age: 35-54 (ref = 18-34) | -0.09 | 0.08 | 0.266 |
|  | **Age: 55+ (ref = 18-34)** | **-0.48** | **0.17** | **0.006** |
|  | Distance to nearest monitoring station (log) | -0.03 | 0.04 | 0.490 |
|  |  | F | EDF | p-value |
| Non-parametric smooth | **Avg. PM2.5 on smoke days: 18-34** | **6.41** | **2.08** | **0.002** |
|  | **Avg. PM2.5 on smoke days: 35-54** | **7.70** | **2.41** | **< 0.001** |
|  | Avg. PM2.5 on smoke days: 55+ | 3.17 | 1 | 0.075 |
|  | Avg. PM2.5 on non-smoke days | 0.09 | 1 | 0.765 |

**Fig. S1.** Nonlinear slopes for average PM2.5 on smoke days across education levels. Results derived from a generalized additive mixed model using “by” factor splines to model different slopes for different education levels. Shaded intervals are 95% confidence intervals. Dashed lines on the bottom of the plot represent individual datapoints.

**Fig. S2.** Nonlinear slopes for average PM2.5 on smoke days across gender levels. Results derived from a generalized additive mixed model using “by” factor splines to model different slopes for different gender levels. Shaded intervals are 95% confidence intervals. Dashed lines on the bottom of the plot represent individual datapoints.

**Fig. S3.** Nonlinear slopes for average PM2.5 on smoke days across income levels. Results derived from a generalized additive mixed model using “by” factor splines to model different slopes for different income levels. Shaded intervals are 95% confidence intervals. Dashed lines on the bottom of the plot represent individual datapoints.

**Fig. S4.** Nonlinear slopes for average PM2.5 on smoke days across health condition levels. Results derived from a generalized additive mixed model using “by” factor splines to model different slopes for different health condition levels. Shaded intervals are 95% confidence intervals. Dashed lines on the bottom of the plot represent individual datapoints.

**Fig. S5.** Nonlinear slopes for average PM2.5 on smoke days across health condition levels. Results derived from a generalized additive mixed model using “by” factor splines to model different slopes for different age levels. Shaded intervals are 95% confidence intervals. Dashed lines on the bottom of the plot represent individual datapoints.
